# Active mRNA degradation by EXD2 nuclease elicits recovery of transcription after genotoxic stress

**DOI:** 10.1038/s41467-023-35922-5

**Published:** 2023-01-20

**Authors:** Jérémy Sandoz, Max Cigrang, Amélie Zachayus, Philippe Catez, Lise-Marie Donnio, Clèmence Elly, Jadwiga Nieminuszczy, Pietro Berico, Cathy Braun, Sergey Alekseev, Jean-Marc Egly, Wojciech Niedzwiedz, Giuseppina Giglia-Mari, Emmanuel Compe, Frédéric Coin

**Affiliations:** 1 Institut de Génétique et de Biologie Moléculaire et Cellulaire Illkirch Cedex, C.U. Equipe Labellisée Ligue contre le Cancer, 2022 Strasbourg, France; 2 Centre National de la Recherche Scientifique, UMR7104 Illkirch, France; 3 Institut National de la Santé et de la Recherche Médicale, U1258 Illkirch, France; 4 Université de Strasbourg, Strasbourg, France; 5 Institut NeuroMyogène (INMG) – Laboratoire Physiopathologie et Génétique du Neurone et du Muscle, Université Claude Bernard Lyon 1, CNRS UMR 5261, INSERM U1315, Lyon, France; 6 The Institute of Cancer Research, London, SW3 6JB UK

**Keywords:** Transcription, Nucleotide excision repair

## Abstract

The transcriptional response to genotoxic stress involves gene expression arrest, followed by recovery of mRNA synthesis (RRS) after DNA repair. We find that the lack of the EXD2 nuclease impairs RRS and decreases cell survival after UV irradiation, without affecting DNA repair. Overexpression of wild-type, but not nuclease-dead EXD2, restores RRS and cell survival. We observe that UV irradiation triggers the relocation of EXD2 from mitochondria to the nucleus. There, EXD2 is recruited to chromatin where it transiently interacts with RNA Polymerase II (RNAPII) to promote the degradation of nascent mRNAs synthesized at the time of genotoxic attack. Reconstitution of the EXD2-RNAPII partnership on a transcribed DNA template in vitro shows that EXD2 primarily interacts with an elongation-blocked RNAPII and efficiently digests mRNA. Overall, our data highlight a crucial step in the transcriptional response to genotoxic attack in which EXD2 interacts with elongation-stalled RNAPII on chromatin to potentially degrade the associated nascent mRNA, allowing transcription restart after DNA repair.

## Abstract (web summary)

Here the authors show that the exonuclease EXD2 is involved in the recovery of class II gene transcription after UV irradiation. EXD2 travels from the mitochondria to the nucleus to interact with RNA Pol II and degrade new synthetized mRNA to allow transcription following DNA repair.

## Introduction

Cells are regularly exposed to endogenous and exogenous genotoxic attacks that induce damage in the DNA molecule^1,2^. The generation of DNA damage can potentially challenge several fundamental cellular processes such as transcription or replication and can ultimately cause diseases such as cancer if not repaired^3–5^. The identification of several protective mechanisms against genotoxic stress highlights the importance of maintaining genome integrity to ensure low mutation frequencies in the genome^6^. One such mechanism, the nucleotide excision repair (NER) pathway, removes DNA adducts such as pyrimidine (6–4) pyrimidone (6–4PP) or cyclobutane pyrimidine dimers (CPD) that are produced by UV light^7–9^. Two NER sub-pathways co-exist in cells: global genome NER (GG-NER), which removes DNA damage from the entire genome, and transcription-coupled NER (TC-NER), which corrects lesions located on actively transcribed genes^8,10–12^. In GG-NER, the concerted action of XPC and/or DDB2-containing complexes enables the detection of DNA damage in the genome, whereas in TC-NER, an actively transcribing RNA Polymerase II (RNAPII), which is stalled by a lesion, triggers efficient removal of cytotoxic damage^13,14^.

To protect the integrity of gene expression under genotoxic attack, cells undergo a transcription stress response that includes global inhibition of transcription occurring in two steps: rapid and local inhibition of elongation due to the stalling of RNAPII in front of transcription-blocking DNA damage^15^ which is followed by a global inhibition of transcription initiation occurring on both damaged and undamaged genes^16,17^. Recent evidence has shown that global inhibition takes place after the degradation of the pool of RNAPII^18,19^. After DNA repair, cells recover transcription in an active process involving transcription and chromatin remodeling factors^20–24^. Recovery of RNA synthesis (RRS) encompasses both the re-initiation of expression at the promoters of actively transcribed genes and the restart of RNAPII molecules already in elongation. Despite recent advances in our understanding of the transcriptional stress response to genotoxic attack, the actors and mechanisms responsible for RRS after DNA repair remain largely elusive. Finding new players involved in RRS is therefore crucial to better understand this process at the molecular level and its role in genome stability.

We unveil here that EXD2, a RNA/DNA nuclease previously shown to be involved in homologous recombination and in the replication fork protection pathway^25,26^, is essential for RRS after the genotoxic attack. Cells lacking EXD2 or expressing a nuclease-dead version of the enzyme are unable to restore global RNAPII-dependent transcription after UV irradiation, resulting in decreased resistance to genotoxic attack. Mechanistically, we demonstrated that EXD2 is not involved in the removal of UV-induced photoproducts. Instead, UV irradiation provokes the re-localization of EXD2 from mitochondria to the nucleus and its translocation to chromatin. There EXD2 transiently interacts with RNAPII and potentially promotes the degradation, during the recovery phase of transcription, of nascent mRNA being synthesized at the time of the genotoxic attack. Using a reconstituted transcription system in vitro, we reconstructed the dynamic association of EXD2 to RNAPII on a transcribed DNA template and demonstrated that EXD2 preferentially interacts with an elongation-blocked RNAPII. In such system, the ribonuclease activity of purified EXD2 efficiently processes mRNA. Accordingly, the interaction between EXD2 and a stalled-RNAPII was also observed in vivo using proximal ligation assay (PLA). These findings unveil a crucial role for EXD2 in the transcription stress response and are the first to assign a nuclear function to the ribonuclease activity of EXD2 by showing its involvement in the degradation of mRNA under synthesis at the time of the genotoxic attack. This degradation is necessary for an efficient recovery of gene expression after DNA repair.

## Results

### UV-induced inhibition and recovery of transcription at a defined genomic locus

In order to identify factors required for transcription recovery after a genotoxic attack, we first sought to develop a sensitive assay to easily monitor transcription inhibition and recovery after UV irradiation. As depicted in Supplementary Fig. 1a, we used a doxycyclin (dox)-inducible transcription/translation reporter system integrated at a single site on genomic DNA in the human osteosarcoma U-2 OS cell line. This system allows visualization of the genomic locus, its nascent mRNA transcript (CFP-SKL), and protein product (CPF-SKL)^27,28^. After a 2-h dox treatment, we detected transcription of CFP-SKL in 80% of the cells (Supplementary Fig. 1b). A 2-h dox treatment followed by a recovery period in the absence of dox (1- to 4-h) triggered an accumulation of CFP-SKL protein expression (Supplementary Fig. 1c). The plasticity of this system also allowed us to measure the transcriptional activity of the cells in a specific time window after UV-irradiation. For this purpose, cells were irradiated with UV (30 J/m^2^) and pulsed with dox for 2 h at different times after the genotoxic attack. Under these conditions, we noticed a strong inhibition of CPF-SKL mRNA expression at early times after UV irradiation (Supplementary Fig. 1d, right panel, lanes 1 and 2). Interestingly, CPF-SKL mRNA expression recovered over time after irradiation (lane 3) and knockdown of the TC- and GG-NER factor XPA prevented this recovery (lanes 4–6 and left panel). CPF-SKL protein expression followed that of its mRNA with a strong inhibition early after irradiation and a progressive recovery that was completed 18 h after irradiation (Supplementary Fig. 1e). These data indicate that CPF-SKL expression recapitulates the rapid inhibition and progressive recovery of global transcription that is generally completed 20 h after a genotoxic attack when DNA repair is efficient^29,30^.

### EXD2 is a critical factor for RRS

**Fig. 1:**
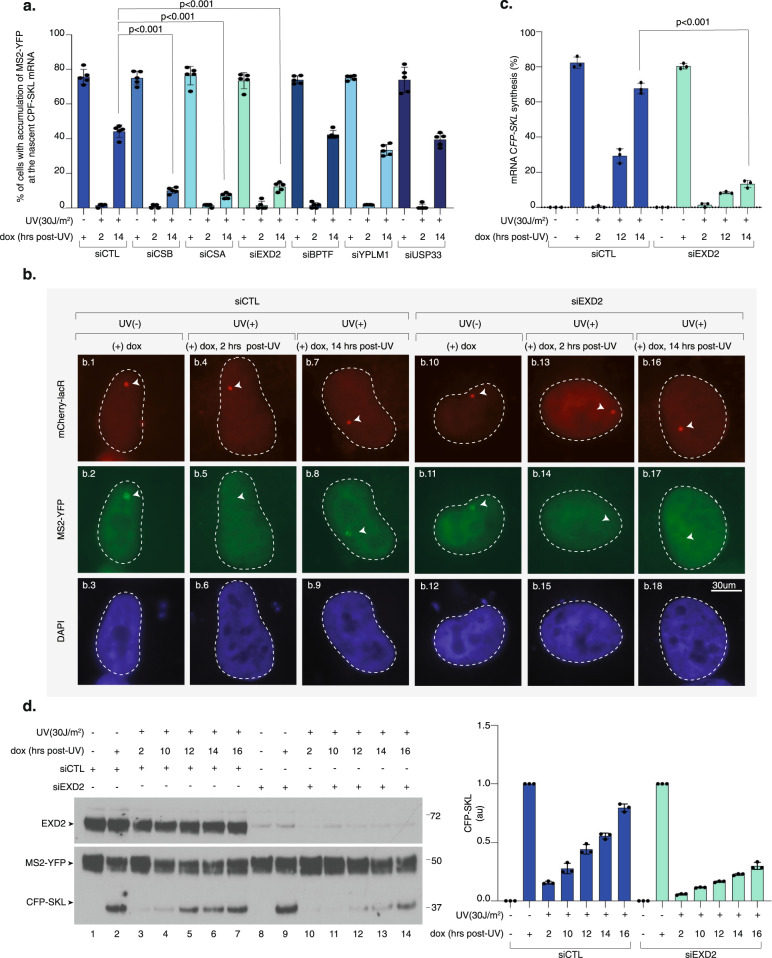
Knockdown of EXD2 impairs RRS of CFP-SKL. **a** U-2 OS cells were transfected with siRNA for 24 h, then with a construct expressing mCherry-lacR for 24 h before UV irradiation (30 J/m^2^) and 2-h pulse-incubation with dox starting at various time points post-UV. Nascent CFP-SKL mRNAs were detected at the reporter locus by accumulation of the MS2-YFP protein to the MS2 RNA loop. Quantification of the transcribing locus is expressed as % of cells showing YFP-MS2 accumulation at a single locus (*n* = at least 250 cells in five independent experiments). Bars represent mean values of three different experiments (Biological triplicates) (+/−SD). One-way ANOVA with post-hoc Tukey adjustment comparisons were used to determine the *p*-values. Source data are provided as a Source Data file. **b** Representative confocal images of cells treated with siCTL or siEXD2. Images of the cells were obtained with the same microscopy system and constant acquisition parameters. **c** Cells were treated as described above and after the 2-h pulse-incubation with dox, the relative amount of CFP-SKL mRNA was quantified by RT-qPCR. Bars represent mean values of three different experiments (Biological triplicates) (+/−SEM). One-way ANOVA with post-hoc Tukey adjustment comparisons were used to determine the *p*-values. Source data are provided as a Source Data file. **d** U-2 OS cells were treated as described above. After the 2-h pulse-incubation with dox, the cells were let to recover for 4 h before lysis. Extracts were resolved by SDS-PAGE and immuno-blotted with anti-GFP (recognizing both the MS2-YFP and CFP-SKL proteins) and anti-EXD2. Lanes 1 and 8 are negative controls in which cells were not treated with dox. Lanes 2 and 9 are positive controls in which cells were treated with dox for 2 h before to recover 4 h in the absence of dox. Molecular sizes are indicated (KDa). CFP-SKL signals were quantified using ImageJ software (NIH), normalized with YFP-MS2 signals and reported on the graph (1 is the value for dox (+) for siCTL or siEXD2). Bars represent mean values of three different experiments (Biological triplicates) (+/−SEM). Source data are provided as a Source Data file.

To explore the mechanism of transcription recovery after genotoxic stress, we used the reporter assay described above and tested a small number of selected candidates for their potential involvement in RRS. As expected, knockdown of the TC-NER factors CSA and CSB inhibited recovery of CFP-SKL expression (Fig. 1a). Interestingly, the 3’ to 5’ DNA/RNA exonuclease EXD2 emerged as a potential effector of transcription recovery after UV irradiation (Fig. 1a, b). The lack of recovery of CFP-SKL expression in the absence of EXD2 was confirmed by RT-qPCR (Fig. 1c) and resulted in 70% inhibition of de novo translation of CFP-SKL over time after UV irradiation (Fig. 1d, compare lanes 10–14 and 3–7). Of note, we observed that the accumulation of nascent CFP-SKL mRNA or de novo CFP-SKL protein was not impaired by the knockdown of EXD2 in the mock-treated cells (Fig. 1b, compare panels b1-b2-b3 with b10-b11-b12 and Fig. 1d, compare lane 2 and 9). These data suggest that EXD2 is required for RRS following a genotoxic stress.

### EXD2 nuclease activity is required for RRS

**Fig. 2:**
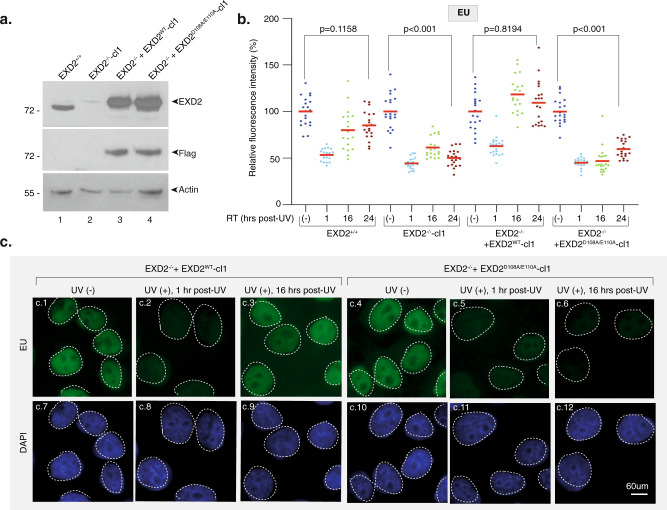
Knockdown of EXD2 nuclease activity impairs global RRS. **a** Protein lysates from EXD2^+/+^, EXD2^−/−^-cl1, EXD2^−/−^ + EXD2^WT^-cl1 and EXD2^−/−^ + EXD2^D108A/E110A^-cl1 cells were immuno-blotted for proteins as indicated. Molecular mass of the proteins is indicated (kDa). Source data are provided as a Source Data file. **b** Cells were mock- or UV-irradiated (15 J/m^2^) and mRNA was labeled with EU at the indicated time points post-UV. EU signal was quantified by ImageJ and relative integrated densities, normalized to mock-treated level set to 100%, are reported on the graph (*n* = at least 20 cells per conditions). Red bars indicate mean integrated density. RT recovery time. One-way ANOVA with post-hoc Tukey adjustment comparisons were used to determine the *p*-values. Source data are provided as a Source Data file. **c** Representative confocal images of EXD2^−/−^ + EXD2^WT^-cl1 and EXD2^−/−^ + EXD2^D108A/E110A^-cl1 treated like in **b**. Images of the cells were obtained with the same microscopy system and constant acquisition parameters.

We next used HeLa EXD2 CRISPR knock-out cells (EXD2^−/−^-cl1)^25^ (Fig. 2a, compare lanes 1 and 2) to measure the impact of EXD2 on global RRS. We pulse-labeled nascent mRNAs at various time points after UV irradiation (15 J/m^2^) using 5-ethynyluridine (EU)^31^. At this UV dose, all transcribed gene strands should contain at least one lesion that blocks RNAPII elongation^32^. We pre-treated the cells with a low concentration of actinomycin D (0.05 μg/ml) to abolish the intense nucleolar EU staining due to RNAPI-dependent ribosomal RNA synthesis. In these conditions, EU incorporation mainly reflects RNAPII-dependent RNA transcription^31^. Within the first hour after UV irradiation, we observed a strong inhibition (50%) of mRNA synthesis in both EXD2^+/+^ and EXD2^−/−^-cl1 cells (Fig. 2b and Supplementary Fig. 2a, panels a.1-a.2 and a.5-a.6). In agreement with the above data, RRS was progressively restored in wild-type EXD2^+/+^ cells over time, whereas it remained deficient in EXD2^−/−^-cl1 cells (Fig. 2b and Supplementary Fig. 2a panels a.3-a.4 and a.7-a.8). This defect was similar to the RRS defect observed in the CS1ANSV cell line from CS-B patient (in which the TC-NER factor CSB was deficient)^33^ (Supplementary Fig. 2b).

To explore the role of the exonuclease activity of EXD2 in RRS, EXD2^−/−^-cl1 cells were subsequently complemented with either FLAG-HA-tagged wild-type (EXD2^−/−^ + EXD2^WT^-cl1) or dominant negative nuclease-dead EXD2 containing two substitutions at positions D108 and E110 (EXD2^−/−^ + EXD2^D108A/E110A^-cl1). These two amino-acids are located in the active site of EXD2 and are known to be essential for its nuclease activity^25,34^. RRS was restored in EXD2^−/−^ + EXD2^WT^-cl1 cells but not in the nuclease-dead EXD2^−/−^ + EXD2^D108A/E110A^-cl1 cells (Fig. 2b, c, compare panels c.1, c.2, c.3 with c.4, c.5, c.6), showing that RRS requires the nuclease activity of EXD2. We noted that the stability of the RPB1 subunit of RNAPII after UV-irradiation was not affected by the depletion of EXD2 or the lack of its exonuclease activity (Supplementary Fig. 2c). As noted above, we also observed that mRNA synthesis was indistinguishable in all four mock-treated HeLa cells, suggesting that EXD2 is not required for RNAPII-dependent transcription in the absence of genotoxic attack (Fig. 2c, panels c.1 and c.4, and Supplementary Fig. 2a, panels a.1 and a.5). Similar results were obtained with an additional set of HeLa clones (EXD2^−/−^-cl2, EXD2^−/−^ + EXD2^WT^-cl2 and EXD2^−/−^ + EXD2^D108A/E110A^-cl2) (Supplementary Fig. 2d–e), but also under conditions in which cells were synchronized at G0-G1 to prevent cell division (Supplementary Fig. 2f). Thus, the knockdown as well as overexpression studies complement one another and establish that EXD2 exonuclease activity has a crucial function in RRS following UV irradiation.

In a second set of experiments, we evaluated the role of EXD2 in response to various treatments provoking transcription arrest without generating DNA damage. We either treated the cells with the transcriptional inhibitor 5,6-dichloro-1-beta-D-ribofuranosylbenzimidazole (DRB) for 30 min^31^ or incubated them for 15 min at 4 °C to block transcription. Following the chase of DRB or the re-incubation at 37 °C, we observed similar transcriptional recovery in EXD2^−/−^ + EXD2^WT^-cl1 and EXD2^−/−^ + EXD2^D108A/E110A^-cl1 (Supplementary Fig. 3). Taken together, these results suggest that EXD2 specifically contributes to the global transcription recovery operating after a genotoxic stress such as UV irradiation.

### Lack of EXD2 nuclease activity leads to mild UV sensitivity

**Fig. 3:**
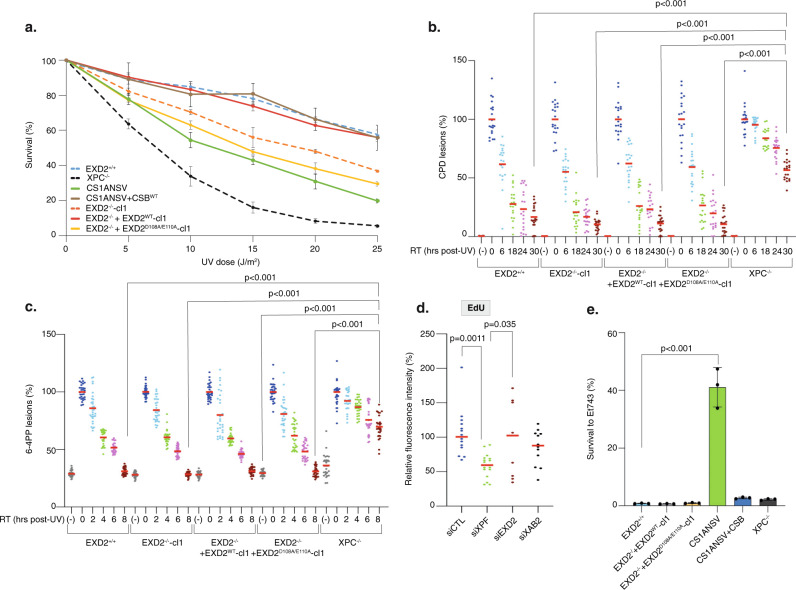
Knockdown of EXD2 nuclease activity sensitizes cells to UV irradiation. **a** Cells were treated with increasing doses of UV irradiation and survival was determined 48 h later. Data were normalized to the mock treatment controls (as value of 100). The values are the means of three independent experiments (+/−SEM) (Technical triplicates). Source data are provided as a Source Data file. **b**, **c** Removal of UV lesions was measured in cells, harvested at different time points post-UV (15 J/m^2^) as indicated. Cells were labeled with anti-CPD (**b**) or anti-6-4PP (**c**) antibodies and signals were quantified using ImageJ at the different times indicated in the figure. Graph represents the % of lesions remaining in the genome at different time points normalized to the amounts of lesions measured immediately after UV irradiation (as a value of 100%). For each time point, a mean of 30 cells has been analyzed. Red bars indicate mean integrated density. RT; recovery time. (-); cells were mock-irradiated. 0; cells were irradiated and fixed immediately. One-way ANOVA with post-hoc Tukey adjustment comparisons were used to determine the *p*-values. Source data are provided as a Source Data file. **d** GG-NER deficient XPC^−/−^ cells were treated either with siRNA against the TC-NER factor XAB2, the TC- and GG-NER factor XPF or against EXD2 for 24 h before local UV irradiation (50 J/m^2^) and EdU staining. The local TCR-UDS signals were quantified by ImageJ and reported on the graph. At least 15 cells were quantified for each situation. Red bars indicate mean integrated density. One-way ANOVA with post-hoc Tukey adjustment comparisons were used to determine the *p*-values. Source data are provided as a Source Data file. **e** Cells were treated with Et743 (0.5 nM) and survival was determined 48 h later. Data were normalized to the mock treatment controls (as a value of 100). The values are the means of three independent experiments (+/−SEM) (Biological triplicates). One-way ANOVA with post-hoc Tukey adjustment comparisons were used to determine the *p*-values. Source data are provided as a Source Data file.

To further examine the consequences of a lack of EXD2 activity on cell homeostasis, we measured the UV sensitivity of EXD2^−/−^-cl1, EXD2^−/−^ + EXD2^WT^-cl1, and EXD2^−/−^ + EXD2^D108A/E110A^-cl1 cells in comparison with the parental EXD2^+/+^ cells as well as the CS-B patient CS1ANSV cell line and the HeLa XPC^−/−^ (in which the GG-NER factor XPC was depleted)^35^. Upon increasing doses of UV irradiation, knockdown of EXD2 activity resulted in hypersensitivity of EXD2^−/−^-cl1 and EXD2^−/−^ + EXD2^D108A/E110A^-cl1 cells, compared to EXD2^+/+^ and EXD2^−/−^ + EXD2^WT^-cl1 cells (Fig. 3a). Interestingly, UV sensitivity of EXD2^−/−^-cl1 and EXD2^−/−^ + EXD2^D108A/E110A^-cl1 cells was similar to that found in the TC-NER deficient CS-B cells but not as pronounced as the one found in the highly sensitive GG-NER deficient XPC^−/−^ cells.

To determine whether EXD2 was involved in the removal of UV-induced DNA damage by NER, we measured GG- and TC-NER in cells depleted of EXD2 activity. To this end, we first performed immunofluorescence-based quantification of UV lesions directly in cell nuclei^31^. The removal rate of the two main types of UV lesions in EXD2^−/−^-cl1 and EXD2^−/−^ + EXD2^D108A/E110A^-cl1 cells was higher to that of HeLa XPC^−/−^ cells and identical to that of EXD2^+/+^ or EXD2^−/−^ + EXD2^WT^-cl1 cells, implying that GG-NER was efficient in cells lacking EXD2 nuclease activity (Fig. 3b, c). We used two different assays to measure TC-NER. We first performed unscheduled DNA repair synthesis (UDS) during TC-NER (TCR-UDS)^36^. Using GG-NER deficient XPC^−/−^ cells to ensure that repair replication in the UV-damage area was due to ongoing TC-NER, we measured repair replication via incorporation of EdU into newly synthesized DNA after local UV irradiation. Loss of the TC-NER specific factor XAB2 or TC/GG-NER factor XPF using siRNA knockdown induced similar deficiency in TCR-UDS, while loss of EXD2 had no impact (Fig. 3d). We next used the particularity of TC-NER deficient cells to be resistant to the DNA binder and anti-cancer drug Ecteinascidin 743 (Et743)^37^. Indeed, the TC-NER deficient CS1ANSV cells showed high resistance to Et743 that was abolished in the recovered CS1ANSV + CSB cells (Fig. 3e). In contrast, knockdown of EXD2 exonuclease activity did not impact the sensitivity of the corresponding cells to Et743. Finally, while γH2AX accumulated after UV-irradiation and persisted in NER deficient cells^38^, no accumulation of γH2AX after knockdown of EXD2 was observed over time after UV irradiation (Supplementary Fig. 4). Altogether, these results suggest that while the knockdown of EXD2 sensitizes cells to UV irradiation, the nuclease is not involved in GG- or TC-NER.

### EXD2 degrades nascent mRNA under synthesis at the time of UV irradiation

**Fig. 4:**
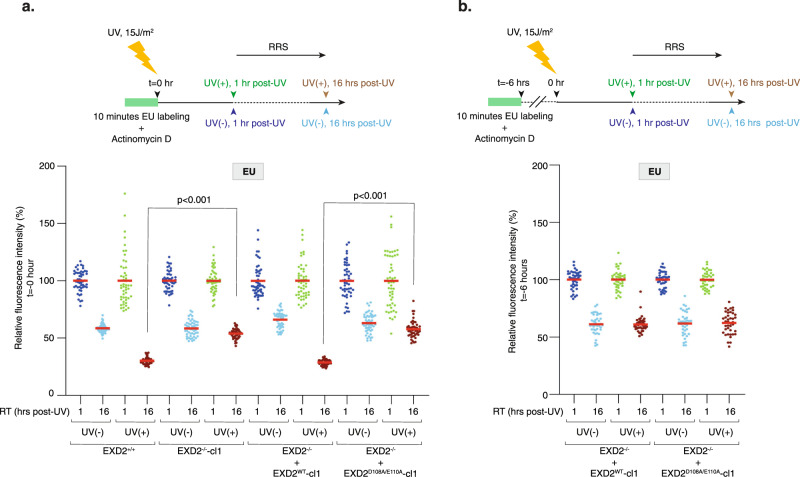
EXD2 degrades mRNA under synthesis at the time of UV irradiation. **a** Upper panel; Scheme of the EU pulse-chase method used to analyze the fate of mRNA being synthesized at the moment of UV irradiation. Cells were incubated for 30 min with Actinomycin D (0.05 μg/ml) to specifically inhibit RNAPI transcription and mRNAs were pulse-labeled with EU for 10 min prior to UV irradiation (15 J/m^2^). Cells were let to recover for 1 h or 16 h post-UV before fixation. Actinomycin D was maintained during the experiment. Lower panel; Cells were treated as indicated in the upper panel and EU signals were quantified using ImageJ and normalized to the value obtained at 1 h set to 100%. Values are reported on the graph (*n* = at least 50 cells). Red bars indicate mean integrated density. RT; recovery time. One-way ANOVA with post-hoc Tukey adjustment comparisons were used to determine the *p*-values. Source data are provided as a Source Data file. **b** Upper panel; Compare to panel **a**, UV irradiation (15 J/m^2^) was performed 6 h after EU labeling and cells were let to recover for either 1 h or 16 h post-UV before fixation. Actinomycin D was maintained during the experiment. Lower panel; Cells were treated as indicated in upper panel and EU signals were quantified using ImageJ. Values are reported on the graph (*n* = at least 50 cells). Red bars indicate mean integrated density. RT recovery time. One-way ANOVA with post-hoc Tukey adjustment comparisons were used to determine the *p*-values. No statistically significant differences were detected at 16 h post-UV between EXD2^−/−^ + EXD2^WT^-cl1 and EXD2^−/−^ + EXD2^D108A/E110A^-cl1 cells. Source data are provided as a Source Data file.

The above data point to a direct processing of mRNA by EXD2 nuclease activity during transcription recovery. To study this function, we first wanted to analyze the fate of mRNA under synthesis at the time of UV irradiation and developed the assay described in Fig. 4a, upper panel. We inhibited ribosomal RNA synthesis with a low concentration of actinomycin D and subsequently labeled nascent mRNAs with a 10 min EU pulse. We then chased EU and immediately UV-irradiated the cells (15 J/m^2^). Fixing them 1 or 16 h post-chase, we were able to follow, during the recovery phase, the fate of mRNAs under synthesis when cells were subject to a genotoxic attack. In the four mock-treated cells, we observed a 50–40% reduced fraction size of EU-labeled mRNAs between 1 and 16 h of culture (probably reflecting both the turn-over of mRNAs and their dilution during cell division) (Fig. 4a and Supplementary Fig. 5a). Interestingly, UV irradiation of wild-type EXD2^+/+^ and EXD2^−/−^ + EXD2^WT^-cl1 cells provoked a 70% reduced fraction size of EU-labeled mRNAs between 1 and 16 h of culture, while EXD2^−/−^-cl1 and EXD2^−/−^ + EXD2^D108A/E110A^-cl1 cells were refractory to this reduction and showed a situation similar to mock-treated cells with a 50–40% reduced fraction size of EU-labeled mRNAs (Fig. 4a and Supplementary Fig. 5a). Similarly, TCR-deficient CS-B cells were refractory to UV-induced reduction of EU-labeled mRNAs, which was restored after CSB^WT^ expression (Supplementary Fig. 5b). In another set of experiments, we performed UV-irradiation long after EU labeling (6 h) so that the labeled mRNAs were synthetized long before the UV treatment (Fig. 4b, upper panel). In these conditions, the reduced fraction size of labeled mRNAs between 1 and 16 h after irradiation was 50–40% for the four cell lines, regardless of whether EXD2 nuclease activity was present or not, a situation that resembles that of the mock treatment (Fig. 4b). These experiments suggest that the EXD2 nuclease degrades, during the recovery phase, a large fraction of the nascent mRNAs (30%) that were being synthesized at the time of UV irradiation.

### EXD2 translocates to nucleus to interact with RNAPII after UV irradiation

**Fig. 5:**
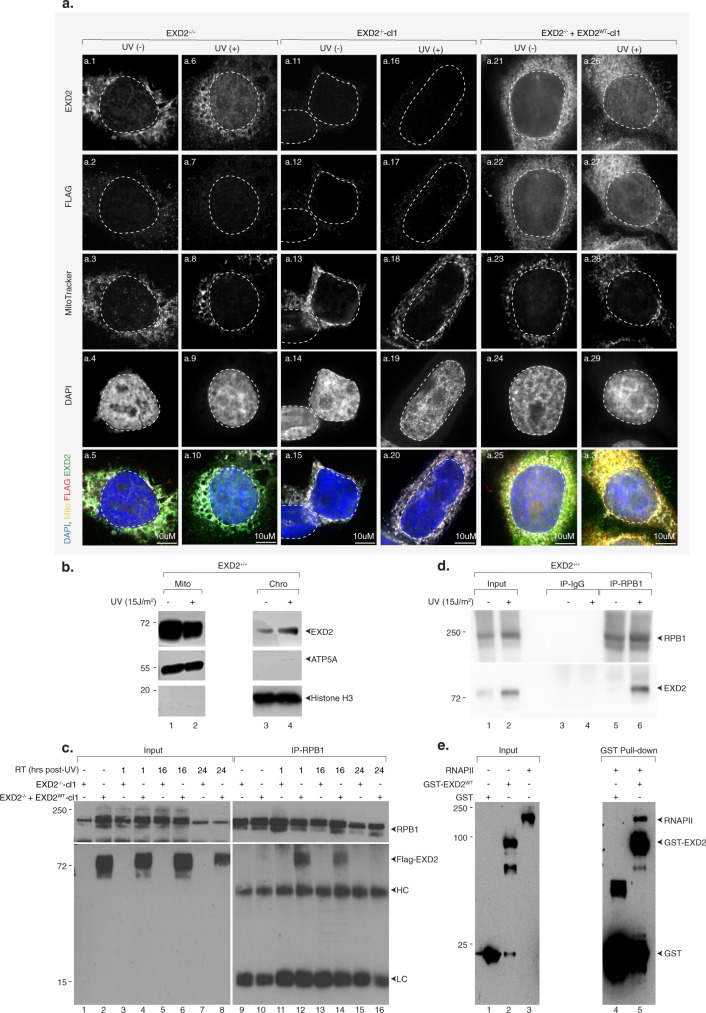
EXD2 transiently interacts with RNAPII after UV irradiation. **a** Representative confocal images of HeLa EXD2^+/+^, EXD2^−/−^ and EXD2^−/−^ + EXD2^WT^-cl1 mock- or UV-irradiated (15 J/m^2^) and left to recover for an hour. Cells were labeled with anti-EXD2 and anti-FLAG and stained with MitoTracker. Images of the cells were obtained with the same microscopy system and constant acquisition parameters for a given labeling/staining. **b** EXD2^+/+^ cells were mock- or UV-irradiated (15 J/m^2^) and let to recover for 1 h. Cells were fractionated in mitochondria (Mito) and chromatin (Chro) fractions, which were resolved by SDS-PAGE and immunoblotted against the indicated proteins. Molecular sizes are indicated (KDa). ATP5A is a marker of mitochondria. Histone H3 is a marker of chromatin. Source data are provided as a Source Data file. **c** EXD2^−/−^-cl1 or EXD2^−/−^ + EXD2^WT^-cl1 cells were mock- or UV-irradiated (15 J/m^2^) and let to recover for the indicated period of time post-UV (RT). RNAPII was immunoprecipitated using anti-RPB1 from total extracts and protein were resolved by SDS-PAGE and immunoblotted using anti-RPB1 or anti-EXD2 antibodies. HC antibody heavy chain, LC antibody light chain, RT recovery time. Molecular sizes are indicated (KDa). Source data are provided as a Source Data file. **d**. RNAPII was immunoprecipitated from chromatin fractions obtained in panel **b**, using anti-RPB1 in the presence of Benzonase. IP using IgG was performed as controls. Proteins were resolved by SDS-PAGE and immunoblotted using anti-RPB1 or anti-EXD2 antibodies. RT recovery time. Molecular sizes are indicated (KDa). Source data are provided as a Source Data file. **e** Purified RNAPII from HeLa cells^39^ was incubated with recombinant pulldown GST-EXD2^WT^. Following washes, fractions were resolved by SDS-PAGE and immunoblotted against the indicated proteins. Controls IP was performed with GST alone (lane 4). Molecular sizes are indicated (KDa). Source data are provided as a Source Data file.

After having established the involvement of EXD2, during the recovery phase, in the degradation of mRNA under synthesis at the time of UV irradiation, we studied the potential connection of the nuclease with RNAPII. Since the literature on EXD2 suggests that the protein has a mitochondrial localization that seems incompatible with a potential nuclear role, we first determined the localization of EXD2 in wild-type EXD2^+/+^ cells in normal conditions and after UV irradiation. Interestingly, whereas the localization of endogenous EXD2 appeared mostly mitochondrial in the absence of genotoxic stress, UV irradiation triggered the re-localization of a large fraction of the endogenous protein from the mitochondria to the nucleus (Fig. 5a, panels a.1-a.10). Similarly, the exogenous flag-tagged EXD2 protein in EXD2^−/−^ + EXD2^WT^-cl1 cells also appeared to partially re-localize to the nucleus after irradiation, although a fraction appeared to localize to the nucleus even in the absence of genotoxic stress (Fig. 5a, panels a.21-a.30). To confirm these observations, we also performed a cell fractionation experiment on EXD2^+/+^ cells. We observed that while EXD2 was mainly localized in mitochondria in the absence of genotoxic stress (although a small fraction was found in the chromatin), a decrease in the amount of EXD2 in this organelle was observed after UV irradiation, coupled with an increase in the amount of EXD2 associated with chromatin (Fig. 5b). Next, we analyzed the potential interaction between EXD2 and RNAPII and its timing in EXD2^−/−^ + EXD2^WT^-cl1 cells. Following UV irradiation (15 J/m^2^), we observed transient coprecipitation between flag-EXD2 and the RPB1 subunit of RNAPII, which was maximal at 1 h after treatment (Fig. 5c, lanes 9–12) and then begins to decrease at later time points to reach the level of mock-treated cells 24 h after UV irradiation (compare lanes 12–14–16). Note that an interaction between endogenous EXD2 and RNAPII was also observed in HeLa EXD2^+/+^, 1 h after UV irradiation (Fig. 5d). We next expressed the full-length GST-tagged EXD2^WT^ in bacteria and performed a GST pull-down assay with purified RNAPII from HeLa cells^39^. GST-EXD2^WT^ pulldown co-precipitated RPB1, suggesting a direct interaction between EXD2 and RNAPII (Fig. 5e). These data highlight a transient direct interaction between EXD2 and RNAPII taking place quickly after UV irradiation and persisting during the recovery phase.

### EXD2 interacts with a subset of RNAPII that stalls persistently on DNA

**Fig. 6:**
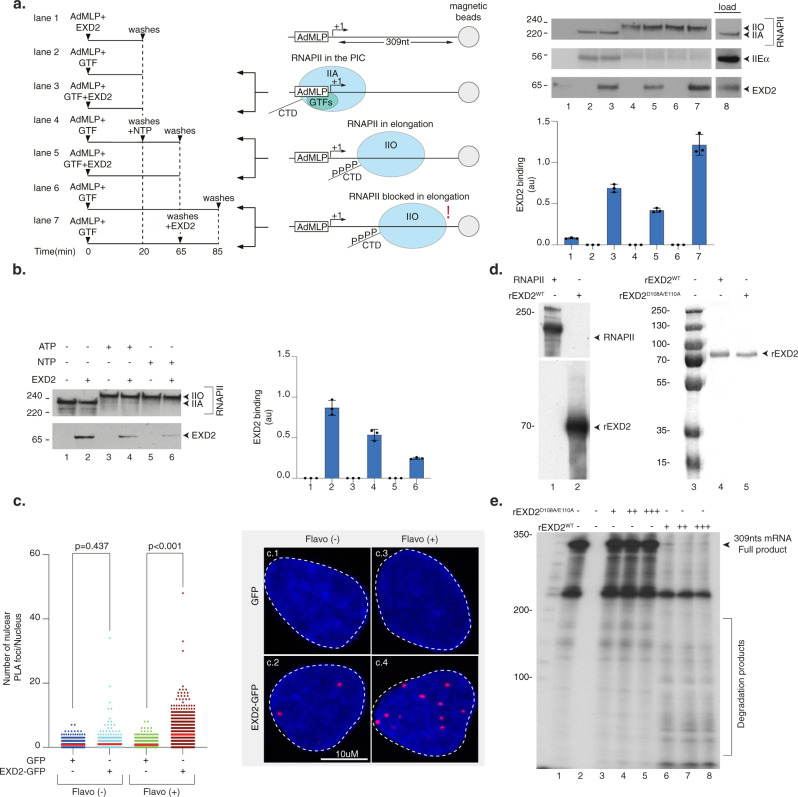
EXD2 preferentially interacts with RNAPII stopped in elongation. **a** Left panel; biotinylated DNA template was bound to streptavidin magnetic beads and incubated for 20 min with purified RNAPII and GTFs. After washes, NTPs were added to initiate RNAPII elongation for 45 min. Middle panel; Three conditions were used: in the absence of NTPs where PIC is formed, in the presence of NTPs in which RNAPII is in elongation, and after the chase of NTPs in which RNAPII is blocked from elongating. Right panel; the binding of different factors was evaluated by immunoblotting. The signals for rEXD2 were quantified and plotted in arbitrary units (au). The values are the means of three independent experiments (+/−SD) (Technical triplicates). Molecular sizes are indicated (KDa). Source data are provided as a Source Data file. **b** The biotinylated DNA template was bound to streptavidin magnetic beads and incubated for 20 min with purified RNAPII and GTFs with or without EXD2 as indicated. After washes, ATP or NTPs were added for 45 min. The binding of different factors was evaluated by immunoblotting and the signals for EXD2 were quantified and plotted in arbitrary units (au). The values are the means of three independent experiments (+/−SD) (Technical triplicates). Molecular sizes are indicated (KDa). Source data are provided as a Source Data file. **c** Number of nuclear GFP/RNAPII PLA foci in U-2 OS cells expressing either GFP or EXD2-GFP with or without Flavopiridol treatment (*n* = at least 100 cells per conditions from three independent experiments). Red bars indicate mean integrated density. One-way ANOVA with post-hoc Tukey adjustment comparisons were used to determine the *p*-values. **d** Left panel; rEXD2^WT^ and purified RNAPII were resolved by SDS-PAGE and immunoblotted with anti-EXD2 and anti-RPB1 antibodies. Molecular sizes are indicated (KDa). Right panel; Coomassie staining of recombinant EXD2^WT^ and EXD2^D108A/E110A^. Lane 3 is the protein markers. Source data are provided as a Source Data file. **e** RNA (309nts) was transcribed from a linear template containing the AdML promoter using a reconstituted RNAPII run-off transcription assay. Then, increasing amount of recombinant EXD2^WT^ or EXD2^D108A/E110A^ (10, 20 and 30 ng) was added to the reaction for an additional 10 min incubation period before the reaction was stopped. Molecular sizes are indicated (Nucleotides). Source data are provided as a Source Data file.

We then asked whether we could reconstitute EXD2 recruitment in vitro on an elongation-blocked RNAPII. We approached this question using a protein/DNA binding assay consisting of a biotinylated DNA template containing the AdMLP promoter and a transcribed region of 309 base pairs. The template was immobilized to streptavidin beads and incubated with purified RNAPII fraction from HeLa cells as well as with the recombinant general transcription factors (GTF: TFIIB, TBP, TFIIE, TFIIF, TFIIH) to form the pre-initiation complex (PIC). Bacterially purified recombinant EXD2 (rEXD2, without GST) was added at different stages of the assay (Fig. 6a, left panel). Addition of NTP induced transcription initiation, whereas their subsequent chase induced RNAPII elongation arrest^40^ (Fig. 6a, middle panel). While western blot analysis of the remaining DNA-bound proteins revealed a very weak background signal of EXD2 to the DNA template in the absence of RNAPII and its GTF (Fig. 6a, right panel, lane 1), a clear recruitment of EXD2 occurred to the PIC in the absence of NTP (lane 3). In contrast, the presence of EXD2 did not improve the recruitment of RNAPII or GTFs (as observed for TFIIEα) (compare lane 2 and 3). The addition of NTP (lane 4) induced the initiation of transcription and the beginning of the elongation step characterized by the emergence of RNAPIIO as well as the release of the basal transcription factor TFIIEα from the DNA template (Fig. 6a, middle panel)^40^. Under these conditions of transcription elongation, EXD2 was released from the RNAPII complex (compare lane 3 with lane 5). Interestingly, the chase of NTP, which blocks RNAPII in elongation, caused EXD2 to be recruited again to the DNA template (compare lane 5 with lane 7). In another set of experiments, we determined whether NTP or ATP were required to induce EXD2 release from RNAPII during initiation. The addition of ATP triggered EXD2 release that was clearly enhanced by the presence of the four NTP (Fig. 6b).

To further demonstrate the association of EXD2 with stalled RNAPII in vivo, we used the proximity-ligation assay (PLA). U-2 OS cells stably expressing C-terminally tagged EXD2-GFP, in which EXD2 localized mainly in mitochondria (Supplementary Fig. 6a), were treated with Flavopiridol, which inhibits RNAPII elongation and causes a slight relocation of EXD2-GFP to the nucleus (Supplementary Fig. 6a, b). After Flavopiridol treatment, we detected a nuclear PLA signal indicating EXD2-RNAPII interaction, which was significantly enriched compared to untreated cells and did not occur with GFP alone (Fig. 6c). These data indicate that EXD2 preferentially interacts with an elongation-blocked RNAPII.

We subsequently examined the impact of the exonuclease activity of EXD2 on newly synthesized mRNA. To detect mRNA, we complemented the above in vitro system with radio-labeled CTP^31^. Note that the purified RNAPII fraction from HeLa cells was devoid of EXD2 (Fig. 6d, left panel). Recombinant human EXD2^WT^ and nuclease-dead EXD2^D108A/E110A^ were purified from insect cells in parallel (Fig. 6d, right panel). Transcription of the AdMLP-containing DNA template led to the production of an mRNA transcript of 309 nucleotides length in 30 min (Fig. 6e, lane 1). The addition of increasing amounts of recombinant purified EXD2^WT^ for the last 10 min of the reaction induced the degradation of the mRNA transcript whereas EXD2^D108A/E110A^ had no impact (compare lanes 3–5 to 6–8). Taken together, these data suggest that a fraction of EXD2 is recruited to chromatin after UV-irradiation to directly interact with elongation-blocked RNAPII and degrade mRNA under synthesis.

## Discussion

Transcription is controlled in time and space by complex epigenetic and signaling-mediated regulatory networks at each step of the process. When cells are subjected to genotoxic attack, DNA damage impacts several crucial cellular functions, including transcription. Indeed, if these lesions are bulky and located in the transcribed strand of an active gene, they become a major complication during its transcription because they constitute a strong barrier to RNAPII forward translocation and result in its blockage, generating transcriptional genotoxic stress^41,42^. Cells cope with this stress firstly by inhibiting global gene expression, then by removing lesions that block RNAPII progression using the TC-NER pathway, and finally by initiating RRS at both promoters and damaged sites. How cells resume transcription after an acute genotoxic attack is crucial because inappropriate restarting is toxic and leads to cellular dysfunction and apoptosis, as observed in cells from patients with CS, which show intermediate sensitivity to UV irradiation coupled with a defect in RRS^43^. With this in mind, we sought to find new players involved in RNAPII-dependent gene expression recovery after genotoxic attack and unveiled a key role for the 3′−5′ exonuclease activity of EXD2 in this process. Recent studies have shown that RNAPI-dependent ribosomal gene transcription is also blocked shortly after a genotoxic stress and recovers over time. A TC-NER machinery removes lesions in ribosomal genes with the participation of CSA and CSB^44^. We tested whether EXD2 was involved in RNAPI-dependent transcription recovery but did not detect a defect in this process in cells lacking EXD2 (Dr. Mari-Giglia, personal information), suggesting a specific involvement of EXD2 in RNAPII-dependent transcription recovery after UV irradiation.

Cells lacking EXD2 nuclease activity exhibited deficient in RRS and intermediate sensitivity to UV irradiation, reminiscent of the phenotype observed in TC-NER deficient cells^10,42^, including CS-B cells in our study. At first glance, this could indicate that EXD2 is involved in TC-NER and in the removal of DNA lesions that block RNAPII during elongation. However, sensitivity to Et743, which required an active TC-NER pathway^37^, and the TCR-UDS assay indicate that TC-NER is unaffected by the absence of EXD2, suggesting an uncoupling of efficient TC-NER from deficient RRS in these conditions. Recently, regulation of the RNAPII pool by ubiquitination was shown to be central in the inhibition and restart of transcription in response to genotoxic stress^18,19^. Persistent depletion of RNAPII was shown to be largely responsible for the lack of transcriptional recovery observed in CS-B cells. Under our conditions, the RNAPII pool was not affected after UV irradiation by the lack of EXD2 nuclease activity, and we ruled out that a direct impact of EXD2 on RNAPII stability was involved in the RRS defect observed in EXD2-deficient cells. Instead, our observations suggest that EXD2 nuclease acts on mRNA, which was observed both in vivo, using pulse-labeling of nascent mRNA, and in vitro, using the transcription/nuclease run-off assay. These assays suggest that the EXD2 nuclease processes, during the recovery phase, a fraction of mRNA representing 30% of the nascent mRNA being synthesized at the time of the genotoxic attack. During RNAPII backtracking, the ability of RNAPII to cleave its transcript potentially allows transcription to resume and cells to survive when the lesions are removed. A plethora of transcription factors, such as TFIIS, are known to stimulate transcript cleavage^45^. Similarly, it seems reasonable to suggest that the exonuclease activity of EXD2 is involved in the mRNA processing associated with RNAPII backtracking in front of DNA lesion as illustrated by the dynamic interaction we observed between them, which transiently kicks in after the genotoxic attack. Moreover, this activity is likely limited to a genotoxic attack situation because mRNA transcription was efficiently recovered after cold-shock treatment in cells lacking EXD2. Our observations also suggest that EXD2 is probably not involved in transcription per se, as EU incorporation or reporter expression was hardly affected in mock-treated cells in our assay and cell viability was largely not affected by EXD2 knockdown in the absence of genotoxic stress (our data and ref. ^25^). This is similar to other genes encoding transcript cleavage stimulatory factors, such a TFIIS, that becomes essential for cell viability only in the presence of a genotoxic stress^46^, which may reflect a potential redundancy in the function of these factors in the absence of stress. The involvement of EXD2 in the recovery of transcription after a genotoxic attack, as well as its role in the repair of double-strand breaks^25,26^ seems to contradict its mitochondrial location^47,48^ that we confirmed in this work. However, our results may potentially reconcile these observations as they suggest that a genotoxic stress such as UV-irradiation leads to a significant re-location of EXD2 from the mitochondria to the nucleus. The molecular aspects of this relocation, and the generalization of this observation to other genotoxic attacks, are not yet known but it suggests post-translational modifications or new protein interactions allowing EXD2 to travel from the mitochondria to the nucleus.

Because EXD2 is known to be a regulator of homologous recombination in double-strand break repair^26^ and double-strand breaks can occur following replication stress, we were also concerned that the lack of RRS might be due to replication stress and not directly to UV irradiation-induced DNA damage. However, we observed first that confluent EXD2-deficient cells synchronized in G0-G1 were also unable to recover transcription after UV irradiation and second that RNAPII-EXD2 interaction occurred when RNAPII is blocked in elongation, even in the absence of genotoxic stress. These data argue for a direct role of EXD2 in transcription recovery in relation to its interaction with RNAPII.

RNAPII backtracking in front of a lesion likely occurs over several nucleotides, such that the 3′ end of the RNA is no longer aligned with the RNAPII active site, preventing transcription restart. Therefore, the data presented here advocate for a scenario in which EXD2, after its re-location to the nucleus, transiently associates with an RNAPII that is stopped persistently on a gene during elongation by the presence of a transcription-blocking lesion, to potentially assist RNAPII in degrading mRNA from 3′ to 5′ when backtracking occurs^41,49^. This activity, alone or in combination with that of RNAPII, could reactivate backtracked RNAPII by providing a new 3′ end to the mRNA to realign RNAPII active site with the ongoing mRNA. Why cells would require the 3′ to 5′ exonuclease activities of RNAPII and EXD2 to process mRNA at a damaged site is unclear, but consistent with this scenario, EXD2 is essential for cell viability after UV irradiation but is not required for NER to occur, demonstrating that UV sensitivity reflects the toxicity of the absence of RRS rather than a defect in DNA damage removal. To better understand the molecular mechanism of EXD2 involvement in RRS, the association of EXD2 with RNAPII was reconstituted in vitro using a transcribed DNA template and highly purified and recombinant transcription factors. Consistent with our hypothesis, we observed that elongation-active RNAPII associated less with EXD2 than elongation-blocked RNAPII on transcribed DNA. It is known that EXD2 discriminates RNA and DNA substrates via metal coordination (Mn2+ vs Mg2+)^34^. We show that under the physicochemical conditions allowing in vitro transcription and the presence of Mg2+, EXD2 exonuclease activity processes newly synthesized long mRNA molecules (309nts length), reinforcing our model of UV-induced recruitment of EXD2 to stalled RNAPII, followed by degradation of nascent mRNA before transcription resumes. Interestingly, these data are also the only ones to assign a ribonuclease function to EXD2 in the nucleus since previous work implicated it in the degradation of nuclear DNA either during DNA double strand break resection in non-homologous end joining or in the protection of stressed replication forks^25,26,50^.

## Methods

### Cell culture

U-2 OS cells were cultured in DMEM (1 g/l Glucose) containing 10% FCS and gentamycin. U-2 OS pTuner 263 cells were cultured in DMEM (1 g/l Glucose) containing 10% Tet-system approved FBS, 1% Penicillin/Streptomycin, 400 μg/ml G418, and 100 μg/ml hygromycin B and 2 μg/ml puromycin. The clones of each cell type (HeLa EXD2^−/−^-cl1 and 2, HeLa EXD2^−/−^ + EXD2^WT^-cl1 and 2 and HeLa EXD2^−/−^ + EXD2^D108A/E110A^-cl1 and 2) were cultured in DMEM (1 g/l Glucose) containing 10% FCS and gentamycin supplemented with 0.25 μg/ml of puromycin for HeLa EXD2^−/−^ + EXD2^WT−^-cl1 and 2 and HeLa EXD2^−/−^ + EXD2^D108A/E110A−^-cl1 and 2. XP4PA-SV, CS1ANSV, CS1ANSV + CSB, and XPC Hela Silencix were cultured as described^33,36,51^ in Dulbecco/HamF10 medium containing 10% FCS. U-2 OS^EXD2−GFP^ and U-2 OS^GFP^ cells were cultured as described^25^ in DMEM medium containing 10% FCS.

### Quantification of actively transcribing cells

U-2 OS pTuner 263 cells were induced by 1 μg/ml of dox for the indicated time intervals. Cells were fixed and the number of cells harboring an YFP-MS2 spot counted.

### CFP-SKL mRNA quantification

Total RNA was purified using TriReagent following the manufacturer’s protocol (Molecular Research Center, TR118) and cDNA was prepared by the SuperScript IV kit (Invitrogen, 18090050). qPCR reactions were carried out using the LightCycler480 (Roche) machine and the LightCycler 480 SYBR Green I Master (Roche, 04887352001).

### EU incorporation assay/RRS assay

RNA labeling by EU incorporation was performed with Click-iT RNA Alexa Fluor 488 Imaging Kit (Invitrogen, C10329) following the manufacturer protocol with the following modifications; 5EU was used at 0.1 mM and labeling was performed during 1 h (with the exception of the RNA degradation assay in which mRNAs were labeled during 10 min) to obtain a good linear EU signal as a function of the incubation time^31^. Microscopy pictures were taken with Leica DM 4000 B equipped with a CoolSnap FX monochrome camera and EU signal intensity was quantified by ImageJ software.

### Immunofluorescence-based DNA lesion quantification

Cells were plated in a 24-well plate. 24 h later, cells were UV-irradiated with UV-C lamp (15 J/m^2^) and recovered for different recovery time intervals at 37 °C, 5% CO_2_. Immuno-labeling of cyclobutane pyrimidine dimers (CPD) and 6–4 photoproducts (6–4PP) was performed using mouse anti-CPD and anti-6-4PP antibodies. DNA was denatured with 2 M HCl for 20 min at RT and blocked in 10% FCS in PBS for 30 min prior to labeling. Microscopy pictures were taken with Leica DM 4000 B equipped with a CoolSnap FX monochrome camera and EU signal intensity was quantified by ImageJ software to determine the percentage of CPD and 6–4PP removal (100% represents the % of lesions measured just after UV irradiation).

### Immunofluorescence

Twenty-four hours after plating, cells were irradiated with UV-C lamps (15 J/m^2^) and recovered for 1 h at 37 °C, 5% CO_2_. Thirty minutes before fixation, cells were treated with MitoTracker Red CMXRos according to the manufacturer’s instructions. After permeabilization (0.02% Triton X-100 for 10 min) and blocking (4% bovine serum albumin, 20 min), cells were immuno-labeled to visualize either endogenous or tag-FLAG EXD2 using rabbit anti-EXD2 (HPA005848, 1/2000) and mouse anti-FLAG M2 antibodies. Slides were mounted with Vectashield containing DAPI. Microscopy pictures were taken with Leica Spinning Disk CSU-W1 and processed with ImageJ software.

### Proximity-ligation assay

U-2 OS cells stably expressing GFP or EXD2-GFP were treated with Flavopiridol (1 μM for 1 h) before permeabilization in 0.5% Triton in PBS for 10 min at 4 °C followed by two washes with PBS, fixation with 3% formaldehyde, 2% sucrose in PBS for 10 min at room temperature and two washes with PBS. Blocking, primary antibody incubation and the PLA assay were then carried out as described^25^. Antibodies employed for the PLA assay were as follows: GFP (Roche, 11814460001, 1:500) and RNAPII (Bethyl, A300-653A, 1:1000). Images were acquired with Zeiss Axio Observer Z1 Marianas^TM^ Microscope attached with a CSU-W spinning disk unit (built by Intelligent Imaging Innovations (3i)) using a 63x objective. Image analysis was carried out with FIJI (ImageJ) and CellProfiler (Broad Institute) software.

### Transfections

Plasmid transfections were conducted using X-tremeGene DNA Transfection Reagent (Roche) according to the manufacturer’s protocols. siRNA transfections were conducted using Lipofectamine RNAiMAX Transfection Reagent (Invitrogen) according to the manufacturer’s protocols.

### TCR-Unscheduled DNA synthesis (TCR-UDS)

GG-NER-deficient XP4PA-SV cells (XP-C) were grown on 18 mm coverslips. siRNA transfections were performed 24 h and 48 h before TCR-UDS assays. After local irradiation at 50 J/m^2^ with UV-C through a 5 µm pore polycarbonate membrane filter, cells were incubated for 8 h with 5-ethynyl-2′-deoxyuridine (EdU), fixed and permeabilized with PBS and 0.5% triton X-100. Then, cells were blocked with PBS + solution (PBS containing 0.15% glycine and 0.5% bovine serum albumin) for 30 min and subsequently incubated for 1 h with mouse monoclonal anti-γH2AX antibody 1:500 diluted in PBS. After extensive washes with PBS containing 0.5% Triton X100, cells were incubated for 45 min with secondary antibodies conjugated with Alexa Fluor 594 fluorescent dyes (Molecular Probes, 1:400 dilution in PBS). Next, cells were washed several times and then incubated for 30 min with the Click-iT reaction cocktail containing Alexa Fluor Azide 488. After washing, the coverslips were mounted with Vectashield containing DAPI (Vector). Images of the cells were obtained with the same microscopy system and constant acquisition parameters. Images were analyzed as follows using ImageJ and a circle of constant size for all images: (i) the background signal was estimated in the nucleus (avoiding the damage, nucleoli and other non-specific signal) and subtracted, (ii) the locally damaged area was defined by using the yH2AX staining, (iii) the mean fluorescence correlated to the EdU incorporation was then measured and thus an estimate of DNA synthesis after the repair was obtained. For each sample, three independent experiments were performed.

### Cell sub-fractionation and IP

Cell fractionation was performed by using the Qproteome Mitochondria Isolation Kit (Qiagen) with some modifications. 2 × 10^7^ HeLa cells were collected 1-h post UV irradiation (20 J/m^2^) and resuspended in cold lysis buffer. Following centrifugation (1000 × *g*, 4 °C), the supernatant was removed and the pellet was resuspended in cold disruption buffer using dounce homogenizer. After centrifugation (1000 × *g*, 4 °C), while the supernatant was treated according to the manufacturer’s instruction to collect mitochondrial fraction, the pellet has been washed three times with PBS and resuspended in sucrose buffer (20mMTris pH 7.6, 15 mM KCl, 60 mM NaCl, 0.34 M sucrose). Under vortex agitation, high salt buffer (20 mM Tris pH 7.6, 25% glycerol, 1.5 mM MgCl_2_, 0.1 mM EDTA, 300 mM NaCl final concentration) was added (30 min at 4 °C). Following centrifugation (2500 × *g*, 4 °C), the supernatant was removed and the pellet (containing the chromatin fraction) was resuspended in sucrose buffer supplemented with 1 mM CaCl_2_. Digestion with Micrococcal Nuclease (25 u, Biolabs) was next performed 5 min at 37 °C and stopped by adding 4 mM EDTA. Sonication was carried out with a Q800R2 sonicator (Qsonica, 3 s on/2 s off during 3 min). The chromatin fraction was collected after centrifugation (16,000 × *g*, 30 min at 4 °C). Bradford protein assays were used to measure the final concentration of the mitochondrial and chromatin fraction. IP experiments were performed with the chromatin fraction using anti-RPB1 monoclonal antibody in the presence or not of Benzonase.

### Protein-DNA binding assay

Biotinylated AdMLP DNA template bound to streptavidin magnetic beads was incubated 20 min at 25 °C with purified RNAPII, TFIIA, IIB, IIF, TBP, IIH and EXD2 in transcription buffer (20 mM HEPES (pH 7.9), 7 mM MgCl_2_, 55 mM KCl). After three washings at 50 mM NaCl, bound fractions were resolved by SDS-PAGE for immunoblottings and others were incubated 45 min at 25 °C with NTP (200 μM). After washings, these fractions were in turn resolved by SDS-PAGE or were further incubated 20 min with EXD2. The abundance of EXD2 was assessed by immunoblot densitometry analysis (using ImageJ software). Each signal was quantified three times and plotted in arbitrary units (au).

### Reconstituted run-off transcription

Reaction mixtures of 12 μL containing 50 ng of linear AdMLP DNA template and recombinant TFIIH, TFIIB, TFIIE, TFIIF, TBP together with purified RNA pol II as described^52^ were pre-incubated for 20 min at 25 °C in transcription buffer (20 mM HEPES (pH7.9), 7 mM MgCl_2_, 55 mM KCl) and transcription was initiated by the addition of 2 μL nucleotide solution to final concentrations of 600 μM UTP, ATP, GTP and 0.6 μM (α-^32^P) CTP. Reactions were carried out for 30 min and recombinant EXD2 was added for another 10 min. Reaction was stopped by the addition of 0.5 μL of 0.5 M EDTA (pH 8). The resulting RNA transcripts were analyzed on an 8% denaturing polyacrylamide gel.

### Statistics and reproducibility

Experimental data were plotted and analyzed using GraphPad Prism (GraphPad Software Inc.). The number of samples and replicates are indicated in the respective figure legends. Each experiment was repeated a least three times with similar results.

### Extended resource table

An extended resource table with antibodies, oligonucleotide sequences, chemicals, and reagents used in this work is provided in Supplementary Table 1.

### Reporting summary

Further information on research design is available in the Nature Portfolio Reporting Summary linked to this article.

## Supplementary information

Supplementary Information

Reporting Summary

## Data Availability

All data generated or analyzed during this study are included in this published article (and its supplementary information files) and are available from the corresponding author on request. An extended resource table with antibodies, oligonucleotide sequences, chemicals and reagents used in this work is provided in Supplemental Table 1. Source data are provided with this paper.

## Source data

Source Data
