# Supplementary material for: Active mRNA degradation by EXD2 nuclease elicits recovery of transcription after genotoxic stress
Source: Nat Commun. 2023 Jan 20;14:341. doi: 10.1038/s41467-023-35922-5 (PMC9859823; doi:10.1038/s41467-023-35922-5)
Supplement: Supplementary file 1 — Supplementary Information [file 41467_2023_35922_MOESM1_ESM.pdf]

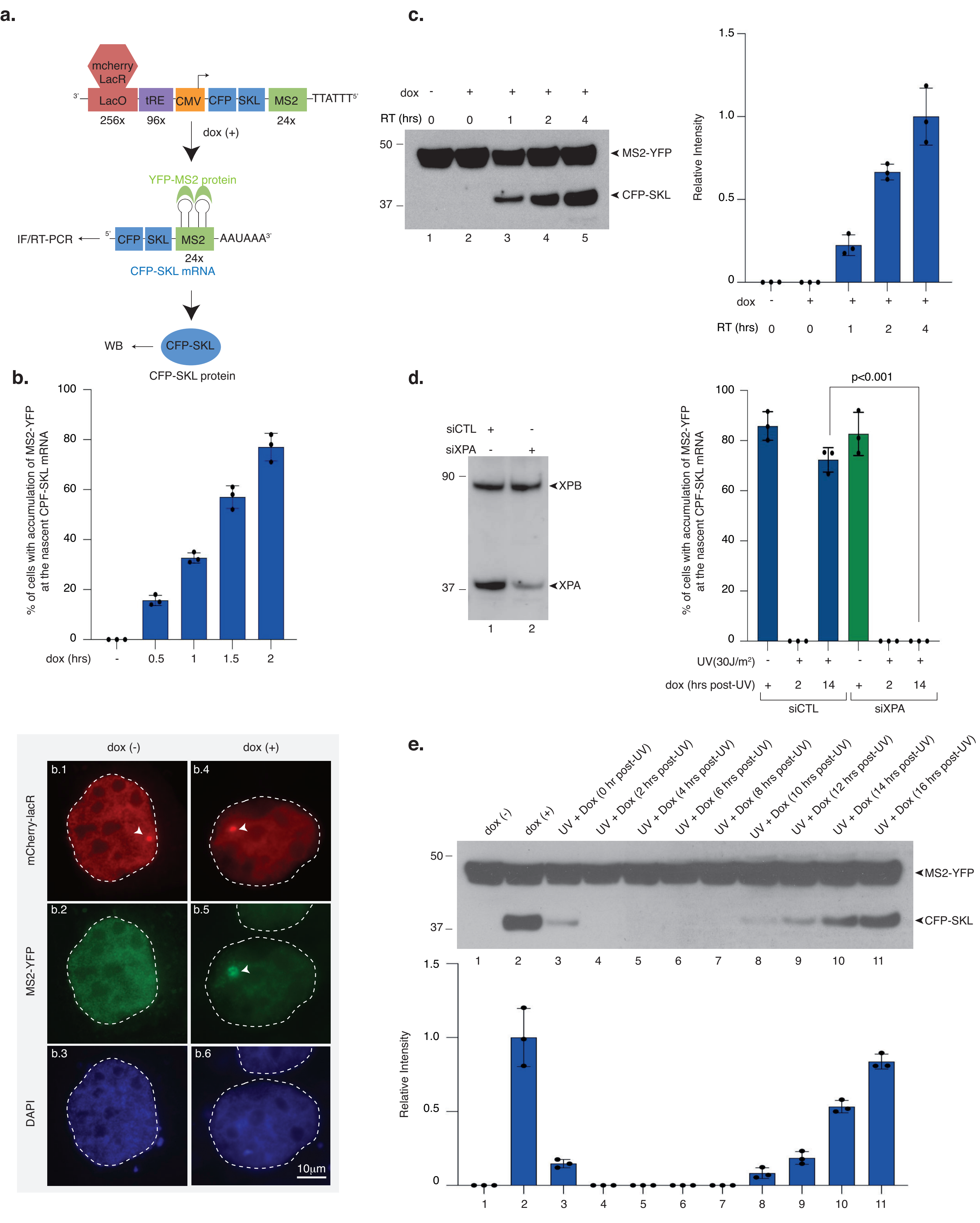
a.
mcherry LacR
LacO
tRE
CMV
CFP
SKL
MS2
TTATTT
256x
96x
24x
dox (+)
YFP-MS2 protein
IF/RT-PCR
CFP
SKL
MS2
AAUAAA
24x
CFP-SKL mRNA
WB
CFP-SKL
CFP-SKL protein
b.
% of cells with accumulation of MS2-YFP at the nascent CPF-SKL mRNA
dox (hrs)
- 0.5 1 1.5 2
dox (-)
dox (+)
mCherry-lacR
MS2-YFP
DAPI
b.1
b.2
b.3
b.4
b.5
b.6
10μm
c.
dox - + + + +
RT (hrs) 0 0 1 2 4
50
37
MS2-YFP
CFP-SKL
1 2 3 4 5
Relative Intensity
dox - + + + +
RT (hrs) 0 0 1 2 4
d.
siCTL + -
siXPA - +
90
37
XPB
XPA
1 2
% of cells with accumulation of MS2-YFP at the nascent CPF-SKL mRNA
p<0.001
UV(30J/m²) - + + - + +
dox (hrs post-UV) + 2 14 + 2 14
siCTL
siXPA
e.
dox (-)
dox (+)
UV + Dox (0 hr post-UV)
UV + Dox (2 hrs post-UV)
UV + Dox (4 hrs post-UV)
UV + Dox (6 hrs post-UV)
UV + Dox (8 hrs post-UV)
UV + Dox (10 hrs post-UV)
UV + Dox (12 hrs post-UV)
UV + Dox (14 hrs post-UV)
UV + Dox (16 hrs post-UV)
50
37
MS2-YFP
CFP-SKL
1 2 3 4 5 6 7 8 9 10 11
Relative Intensity
1 2 3 4 5 6 7 8 9 10 11

Supplementary Figure 1: The CFP-SKL reporter assay

a. 256 copies of lacO array provide examination of the reporter through a mCherry protein fused to lacR. Tetracycline response elements (TREs) control expression of the unique *CFP-SKL* mRNA transcript upon dox treatment. *CFP-SKL* transcription is detected through the binding of YFP-MS2 and encoded CFP-SKL protein that can be detected by WB.

b. U-2 OS were treated with dox as indicated and *CFP-SKL* mRNA expression was quantified. Results are expressed as % of cells showing YFP-MS2 accumulation at a single locus (n= 3x20 cells, biological triplicates). Bars represent mean values of three different experiments (Biological triplicates) (+/- SD). Representative confocal images of MS2-YFP accumulation at *CFP-SKL* is shown. The reporter locus is detected with a mCherry-LacR fusion construct.

c. Immuno-blot for CFP-SKL and YFP-MS2 in U-2 OS cells treated with dox and subsequently let to recover in dox-free medium. Extracts were immuno-blotted with anti-GFP. CFP-SKL signals were normalized with YFP-MS2 signals (+/-SEM). Three independent immunoblots were performed (technical triplicates). Source data are provided as a Source Data file.

d. U-2 OS cells were transfected with siCTL or siXPA for 24 hours and with a construct expressing mCherry-lacR for 24 hours before UV irradiation (30J/m^2) and subsequent 2-hour pulse-incubation with dox. **Left panel**; cell extracts were immunoblotted against XPB and XPA. **Right panel**; Newly transcribed *CFP-SKL* were detected at the reporter locus. Quantification of the transcribing locus was done and results are expressed as % of cells showing YFP-MS2 accumulation at a single locus (n= 3x20 cells, biological triplicates). Bars represent mean values of three different experiments (Biological triplicates) (+/- SD). One-way ANOVA with post-hoc Tukey adjustment comparisons were used to determine the p-values**.** Source data are provided as a Source Data file.

e. U-2 OS cells were UV-irradiated (30 J/m^2) and pulse-incubated for 2 hours with dox. Cells were allowed to recover for 4 hours in the absence of dox. Extracts were immuno-blotted with anti-GFP antibody. Lane 1; cells were not treated with dox. Lane 2; cells were treated with dox for 2 hours before to recover 4 hours in the absence of dox. CFP-SKL signals were normalized

with YFP-MS2 signals (+/-SEM). Three independent immunoblots were performed (technical triplicates). Source data are provided as a Source Data file.

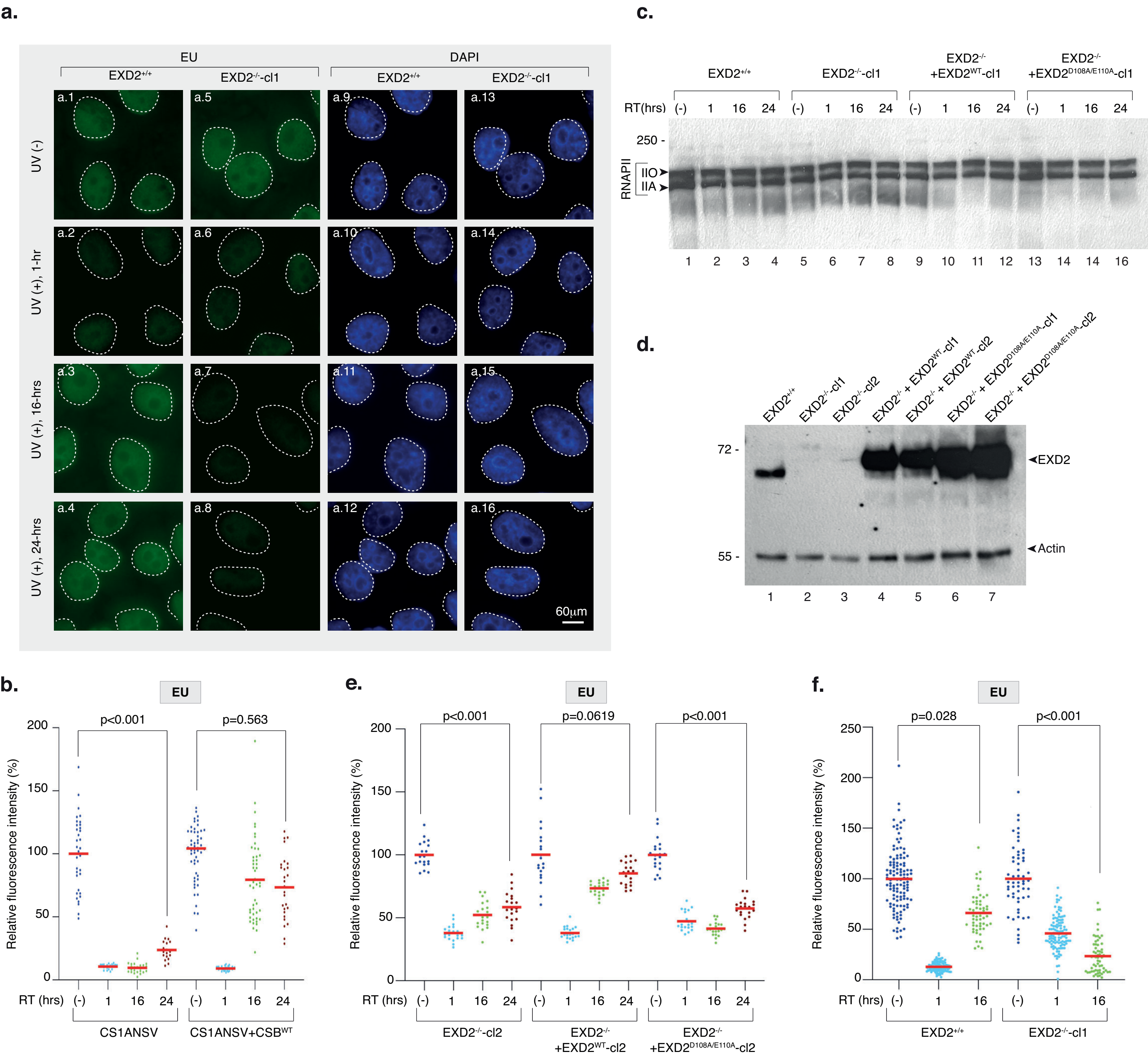
a.
EU
DAPI
EXD2+/+
EXD2-/--cl1
UV (-)
UV (+), 1-hr
UV (+), 16-hrs
UV (+), 24-hrs
a.1 a.2 a.3 a.4 a.5 a.6 a.7 a.8 a.9 a.10 a.11 a.12 a.13 a.14 a.15 a.16
60μm
c.
EXD2+/+
EXD2-/--cl1
EXD2-/- +EXD2WT-cl1
EXD2-/- +EXD2D108A/E110A-cl1
RT(hrs) (-) 1 16 24 (-) 1 16 24 (-) 1 16 24 (-) 1 16 24
250 -
RNAPII
IIO
IIA
1 2 3 4 5 6 7 8 9 10 11 12 13 14 14 16
d.
EXD2+/+
EXD2-/--cl1
EXD2-/--cl2
EXD2-/- + EXD2WT-cl1
EXD2-/- + EXD2WT-cl2
EXD2-/- + EXD2D108A/E110A-cl1
EXD2-/- + EXD2D108A/E110A-cl2
72 -
55 -
EXD2
Actin
1 2 3 4 5 6 7
b.
EU
p<0.001
p=0.563
Relative fluorescence intensity (%)
200 150 100 50 0
RT (hrs) (-) 1 16 24 (-) 1 16 24
CS1ANSV
CS1ANSV+CSBWT
e.
EU
p<0.001
p=0.0619
p<0.001
Relative fluorescence intensity (%)
200 150 100 50 0
RT (hrs) (-) 1 16 24 (-) 1 16 24 (-) 1 16 24
EXD2-/--cl2
EXD2-/- +EXD2WT-cl2
EXD2-/- +EXD2D108A/E110A-cl2
f.
EU
p=0.028
p<0.001
Relative fluorescence intensity (%)
250 200 150 100 50 0
RT (hrs) (-) 1 16 (-) 1 16
EXD2+/+
EXD2-/--cl1

Supplementary Figure 2: Lack of EXD2 induces inhibition of RRS

a. Representative confocal images of $EXD2^{+/+}$ and $EXD2^{-/-}$-cl1. Cells were mock or UV-irradiated (15J/m^2) and mRNA was labelled with EU 1, 16 and 24 hours after UV-irradiation. Images of the cells were obtained with the same microscopy system and constant acquisition parameters.

b. CS1ANSV and CS1ANSV+CSB^{WT} were UV-irradiated (15J/m^2) and mRNA was labelled with EU at 1, 16 and 24 hours after UV-irradiation. EU signal was quantified by ImageJ and relative integrated density normalized to mock-treated level set to 100% are reported on the graph (n=at least 20 cells per condition). Red bars indicate mean integrated density. One-way ANOVA with post-hoc Tukey adjustment comparisons were used to determine the p-values**.** Source data are provided as a Source Data file.

c. HeLa were mock or UV-irradiated (15J/m^2) and let to recover for the indicated times before lysis. Protein lysates were immuno-blotted for RPB1 subunit of RNAPII. Molecular mass of the proteins is indicated on the left (kDa). RT; recovery time. IIO, phosphorylated form of RPB1. IIA non-phosphorylated form. Source data are provided as a Source Data file.

d. Protein lysates were immuno-blotted for proteins as indicated. Molecular mass of the proteins is indicated (kDa). Source data are provided as a Source Data file.

e. HeLa cells were mock or UV-irradiated (15J/m^2) and mRNA was labelled with EU 1, 16 and 24 hours after UV-irradiation. EU signal was quantified by ImageJ and relative integrated density normalized to mock-treated level set to 100% are reported on the graph (n=at least 20 cells per condition). Red bars indicate mean integrated density. One-way ANOVA with post-hoc Tukey adjustment comparisons were used to determine the p-values**.** Source data are provided as a Source Data file.

f. $EXD2^{+/+}$ and $EXD2^{-/-}$-cl 1 cells were grown to 80% confluence and further incubated 24 hours in medium containing 0.5% FCS. Cells were mock or UV-irradiated (15J/m^2) and mRNA was labelled with EU at 1 and 16 hours after UV-irradiation. EU signal was quantified by ImageJ and relative integrated density normalized to mock-treated level set to 100% are reported on the graph (n= at least 50 cells per condition). Red bars indicate mean integrated density. One-

way ANOVA with post-hoc Tukey adjustment comparisons were used to determine the p-values. Source data are provided as a Source Data file.

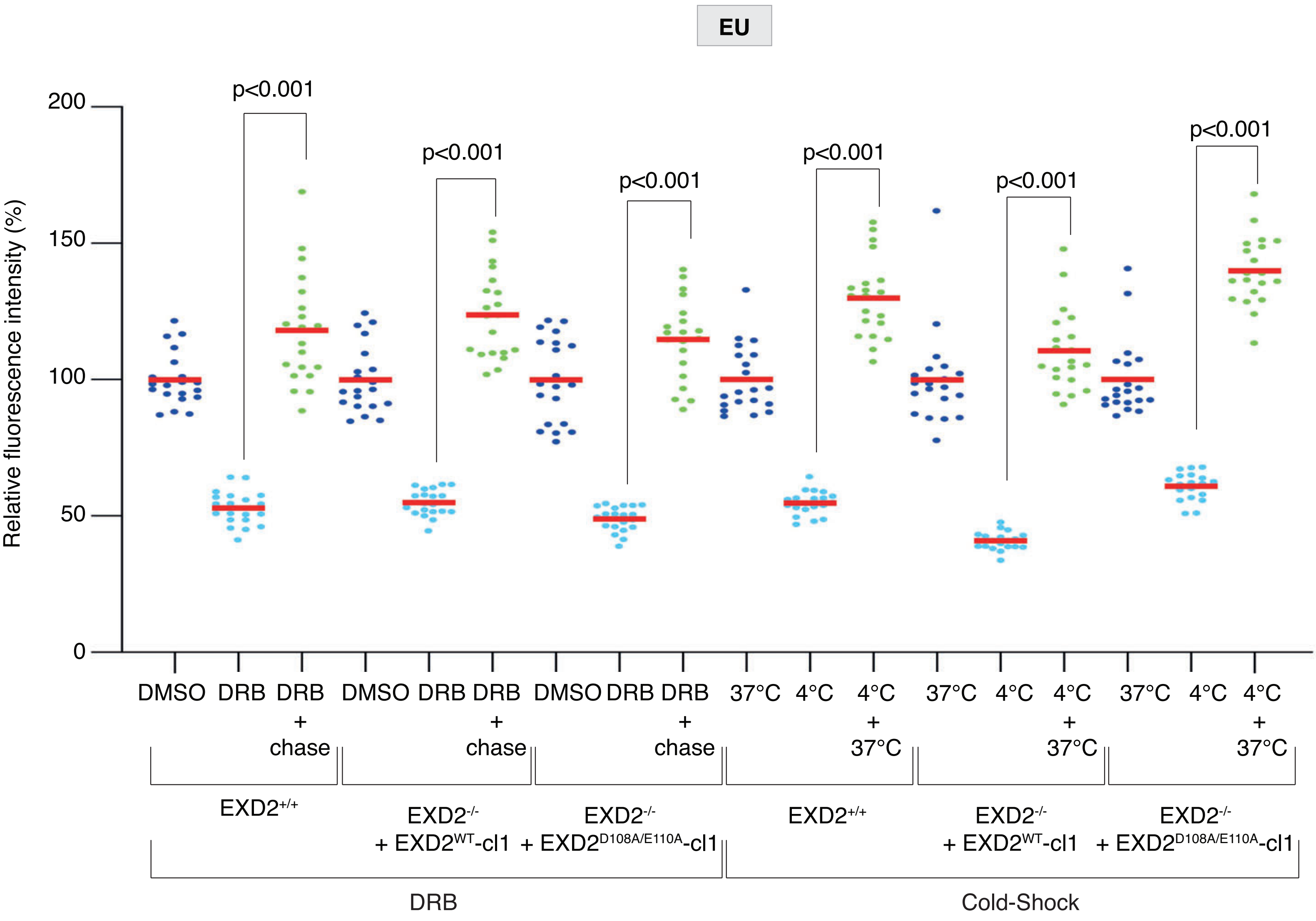
EU
Relative fluorescence intensity (%)
200
150
100
50
0
p<0.001
p<0.001
p<0.001
p<0.001
p<0.001
p<0.001
DMSO DRB DRB + chase
DMSO DRB DRB + chase
DMSO DRB DRB + chase
37°C 4°C 4°C + 37°C
37°C 4°C 4°C + 37°C
37°C 4°C 4°C + 37°C
EXD2+/+
EXD2-/- + EXD2WT-cl1
EXD2-/- + EXD2D108A/E110A-cl1
EXD2+/+
EXD2-/- + EXD2WT-cl1
EXD2-/- + EXD2D108A/E110A-cl1
DRB
Cold-Shock

Supplementary Figure 3: EXD2 is not involved is RRS after DRB or cold-shock treatment

DRB: $EXD2^{+/+}$, $EXD2^{-/-}$ + $EXD2^{WT}$-cl1 and $EXD2^{-/-}$ + $EXD2^{D108A/E110A}$-cl1 were treated with DRB (100 μM) for 1 hour before chase and cells were let to recover for 30 minutes. mRNA was labelled with EU before (DMSO), during (DRB) or after the chase (DRB + chase) of DRB treatment. **Cold-Shock**: $EXD2^{+/+}$, $EXD2^{-/-}$ + $EXD2^{WT}$-cl1 and $EXD2^{-/-}$ + $EXD2^{D108A/E110A}$-cl1 were incubated at 4°C for 15 minutes and let to recover at 37°C for 30 minutes. mRNA was labelled with EU before (37°C), after 15 minutes at 4°C (4°C) or after the recovery at 37°C (4°C+37°C). For both experiments, EU signal was quantified by ImageJ and relative integrated densities normalized to mock-treated level set to 100% are reported on the graph (n= at least 20 cells per condition). Red bars indicate mean integrated density. One-way ANOVA with post-hoc Tukey adjustment comparisons were used to determine the p-values. Source data are provided as a Source Data file.

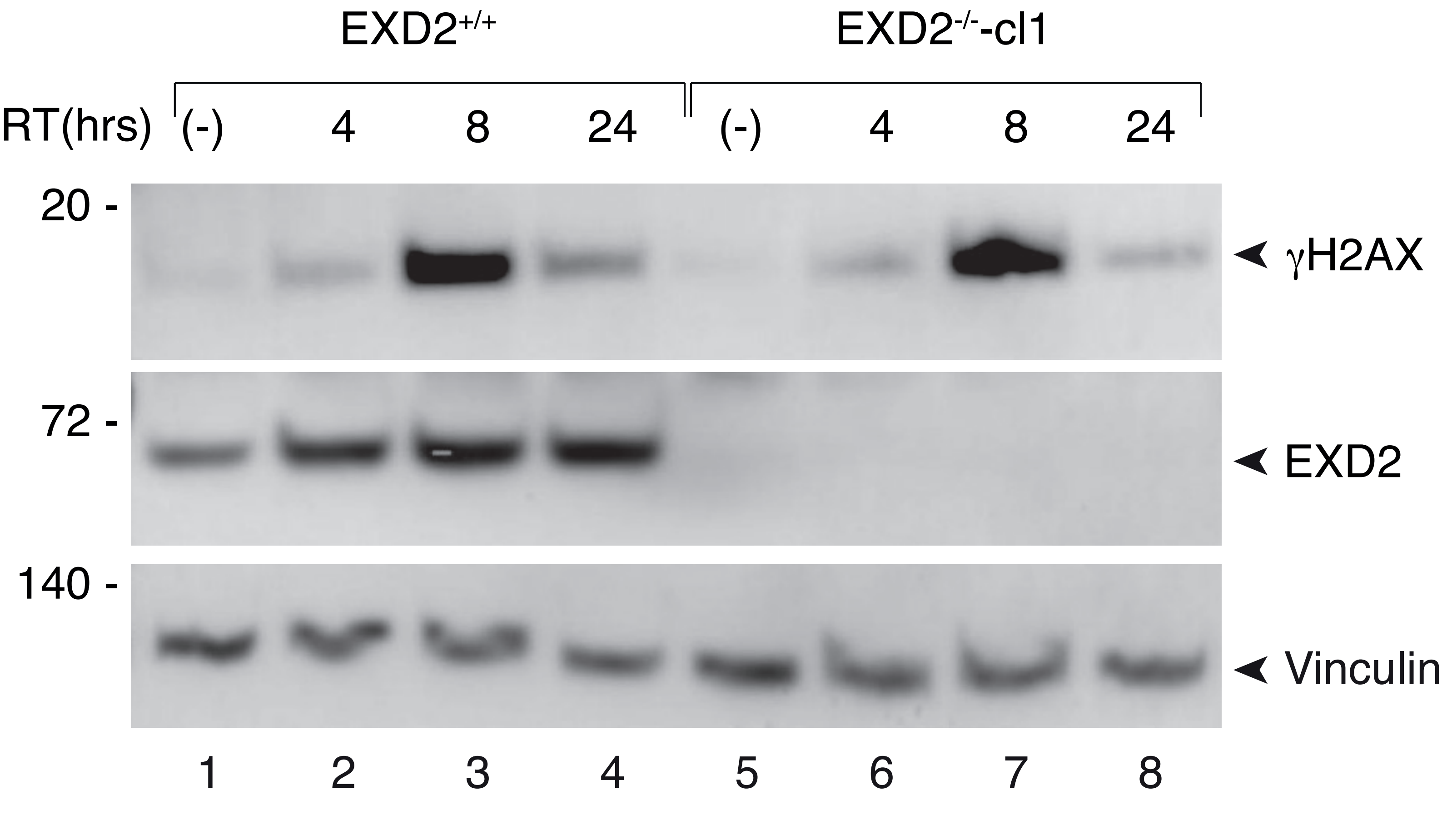
EXD2+/+
EXD2-/--cl1
RT(hrs)
(-) 4 8 24 (-) 4 8 24
20 -
72 -
140 -
γH2AX
EXD2
Vinculin
1 2 3 4 5 6 7 8

Supplementary Figure 4: NER is not affected by the lack of EXD2

EXD2$^{+/+}$ and EXD2$^{-/-}$-cl1 were mock or UV-irradiated (15J/m^2) and let to recover for the indicated times before lysis. Protein lysates were immuno-blotted for EXD2, γH2AX or Vinculin. Molecular mass of the proteins is indicated (kDa). RT; recovery time. Source data are provided as a Source Data file.

a.

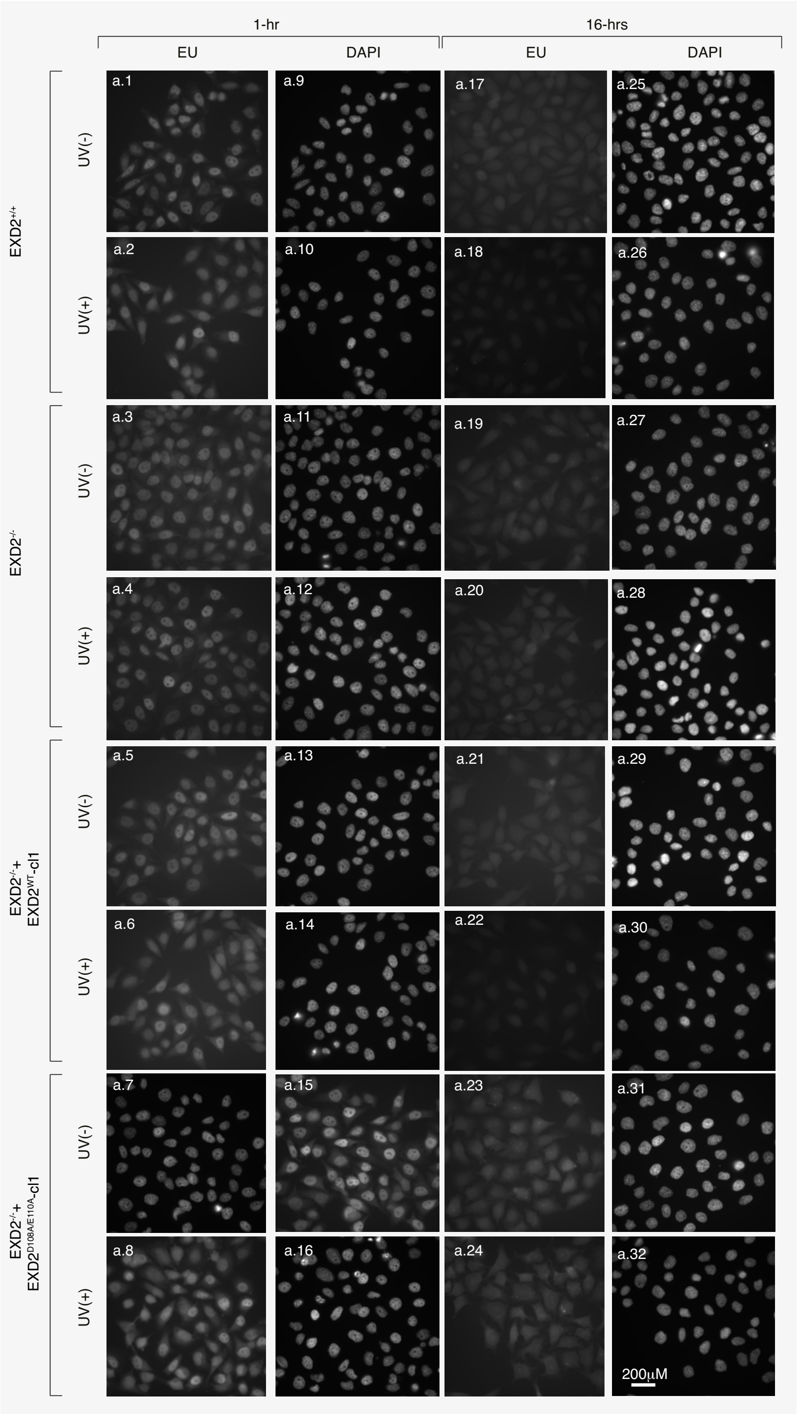
1-hr
16-hrs
EU
DAPI
EU
DAPI
EXD2+/+
EXD2-/-
EXD2-/- +
EXD2WT-cl1
EXD2-/- +
EXD2D108A/E110A-cl1
UV(-)
UV(+)
a.1
a.2
a.3
a.4
a.5
a.6
a.7
a.8
a.9
a.10
a.11
a.12
a.13
a.14
a.15
a.16
a.17
a.18
a.19
a.20
a.21
a.22
a.23
a.24
a.25
a.26
a.27
a.28
a.29
a.30
a.31
a.32
200μM

b.

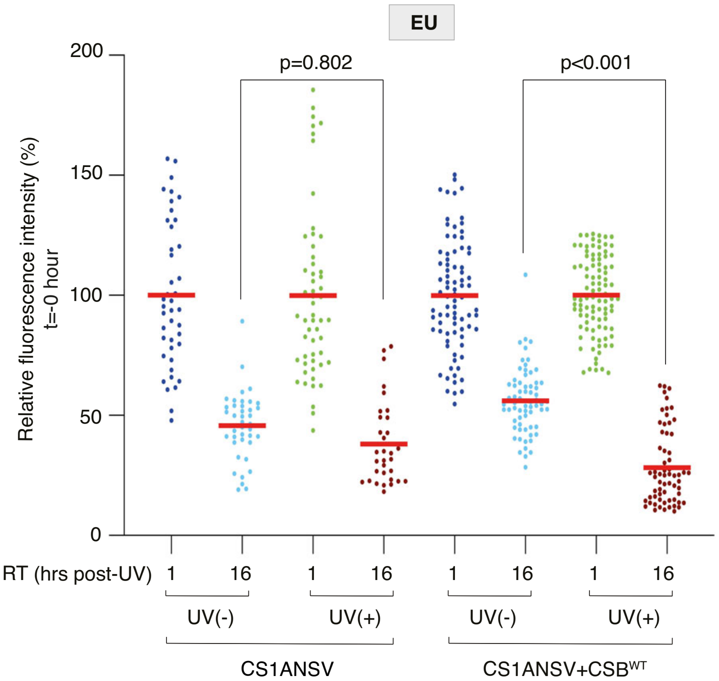
EU
Relative fluorescence intensity (%)
t=0 hour
200
150
100
50
0
p=0.802
p<0.001
RT (hrs post-UV)
1
16
1
16
1
16
1
16
UV(-)
UV(+)
UV(-)
UV(+)
CS1ANSV
CS1ANSV+CSBWT

Supplementary Figure 5: mRNAs under synthesis are degraded by EXD2 following UV irradiation

a. Representative confocal images of $EXD2^{+/+}$, $EXD2^{-/-}$, $EXD2^{-/-}$ + $EXD2^{WT}$-cl1 and $EXD2^{-/-}$ + $EXD2^{D108A/E110A}$-cl1 showing newly synthetized mRNA (1 hour or 16 hours after mock-treatment or UV irradiation at 15J/m^2). Images of the cells were obtained with the same microscopy system and constant acquisition parameters.

b. CS1ANSV and CS1ANSV+CSB^{WT} were treated as indicated in Figure 4a, upper panel, and EU signals were quantified using ImageJ and normalized to the value obtained at 1 hour set to 100%. Values are reported on the graph (n= at least 50 cells). Red bars indicate mean integrated density. *RT*; recovery time. One-way ANOVA with post-hoc Tukey adjustment comparisons were used to determine the p-values. Source data are provided as a Source Data file.

a.

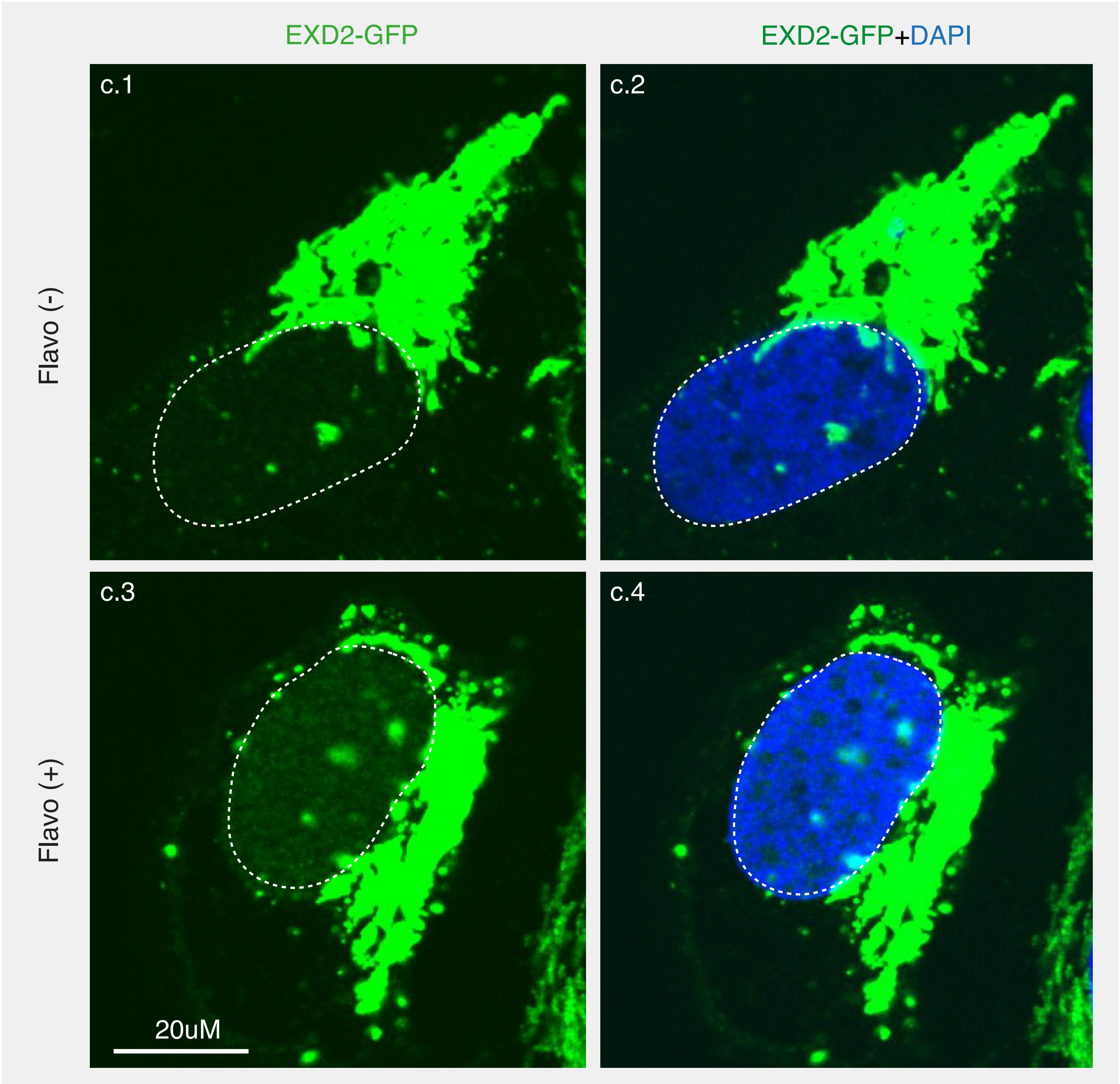
EXD2-GFP
EXD2-GFP+DAPI
c.1
c.2
c.3
c.4
Flavo (-)
Flavo (+)
20uM

b.

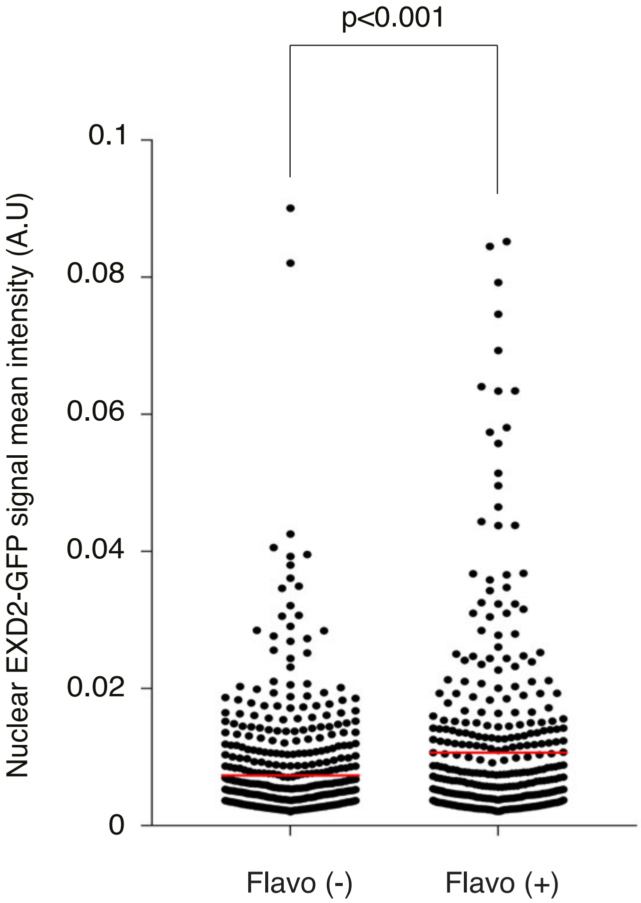
p<0.001
Nuclear EXD2-GFP signal mean intensity (A.U)
0.1
0.08
0.06
0.04
0.02
0
Flavo (-)
Flavo (+)

Supplementary Figure 6: EXD2 ^{WT}-GFP localization in U-2 OS cell

a. Representative confocal images of U-2 OS cells stably expressing EXD2^{WT}-GFP with or without Flavopiridol treatment. Images of the cells were obtained with the same microscopy system and constant acquisition parameters.

b. Quantification of the EXD2-GFP nuclear signal in U-2 OS cells treated or not with Flavopiridol (n= at least 400 cells). Red bars indicate mean integrated density. One-way ANOVA with post-hoc Tukey adjustment comparisons were used to determine the p-values. Source data are provided as a Source Data file.

Supplementary Table 1: Reagents and chemicals

REAGENT or RESOURCE	SOURCE	IDENTIFIER
**Antibodies**		
ACTIN 1/1000	Abcam	AB209856
CPD 1/500	Cosmo Bio	CAC-NM-DND-001
ATP5A 1/1000	ProteinTech Europe	66037-1-IG
EXD2 1/2000	Sigma-Aldrich	HPA005848
FLAG M2 1/1000	Sigma-Aldrich	F1804
GAPDH 1/2000	Abcam	AB8245
GFP 1/2000	Amsbio	TP401
GFP (PLA)	Roche	11814460001
GST 1/1000	Abcam	ab19256
γH2AX 1/1000	Abcam	ab22551
Histone H3 1/5000	Abcam	ab1791100
RPB1 1/5000	Homemade IGBMC	7C2
RNA Pol II (PLA)	Bethyl	A300-653A
TFIIEα 1/1000	Homemade, IGBMC	2A1
Vinculin 1/1000	Sigma Aldrich	V9131
XPA 1/500	SCBT	SC-853
6-4PP 1/1000	Cosmo Bio	CAC-NM-DND-002
**Chemicals, peptides, and recombinant proteins**		
Actinomycin D	Sigma Aldrich	A-1410
DRB	Merck Chemicals	287891
Et743	PharmaMar	(Feuerhahn et al., 2011)
LightCycler 480 SYBR Green I Master	Roche	04887352001
MitoTracker Red CMXRos	Invitrogen	M7512
Streptavidin magnetic beads	Fisher Scientific SAS	88816
TriReagent	Molecular Research Center	TR118
Vectashield	VectorLaboratories	H-1000-10
**Critical commercial assays**		
Click-iT EU Alexa Fluor 488 Imaging Kit	ThermoFisher	C10329
Click-iT EdU Alexa Fluor 488 Imaging Kit	ThermoFisher	C10337
Lipofectamine RNAiMAX Transfection Reagent	Fisher Scientific SAS	13778030
SuperScript IV kit	Invitrogen	18090050
X-tremeGene DNA Transfection Reagent	Roche	063657870001
Qproteome Mitochondria Isolation Kit	Qiagen	37612
**Experimental models: Cell lines**		
U-2 OS and U-2 OS pTuner 263		(Nagy et al., 2016)
CS1ANSV and CS1ANSV+CSB		(Kristensen et al., 2013)
HeLa		Nieminuszczy et al., 2019)
HeLa EXD2$^{-/-}$-cl1 and 2		(Nieminuszczy et al., 2019)

HeLa EXD2$^{-/-}$+EXD2^{WT}-cl1 and 2		(Nieminuszczy et al., 2019)
HeLa EXD2$^{-/-}$+EXD2$^{D108A/E110A}$-cl1 and 2		(Nieminuszczy et al., 2019)
U-2 OS^{GFP}		(Nieminuszczy et al., 2019)
U-2 OS$^{EXD2-GFP}$		(Nieminuszczy et al., 2019)
XP4PA-SV		(Donnio et al., 2019)
XPC HeLa Silencix		(Le May et al., 2010)
**Oligonucleotides**		
F: GCAGAAGAACGGCATCAAGG	*CFP-SKL*	This paper
R: GTCCATGCCGAGAGTGATCC	*CFP-SKL*	This paper
F: CTTCCCCGATGAGAACTTCAAACT	*CycB*	This paper
R: CACCTCCATGCCCTCTAGAACTTT	*CycB*	This paper
siXPF	Dharmacon	019946-00
siXAB2	Dharmacon	004914-01
siEXD2	Dharmacon	020899-02
siCTL	Dharmacon	001810-02
siXPA	Dharmacon	005067-01
siCSB	Dharmacon	004888-00
siCSA	Dharmacon	011008-00
siUSP33	Dharmacon	006081-00
siBPTF	Dharmacon	004025-00
siYLPM1	Dharmacon	032696-00
**Recombinant DNA**		
mCherry-LacR		(Nagy et al., 2016)
**Software and algorithms**		
GraphPad Prism	GraphPad Software Inc.	
ImageJ	NIH	
CellProfiler	Broad Institut	CellProfiler
**Other**		
Leica DM 4000 B equipped with a CoolSnap FX monochrome camera	Leica	

Supplementary References

Donnio, L.-M., Lagarou, A., Sueur, G., Mari, P.-O., Giglia-Mari, G., 2019. CSB-Dependent Cyclin-Dependent Kinase 9 Degradation and RNA Polymerase II Phosphorylation during Transcription-Coupled Repair. Mol Cell Biol 39. https://doi.org/10.1128/MCB.00225-18

Feuerhahn, S., Giraudon, C., Martinez-Diez, M., Bueren-Calabuig, J.A., Galmarini, C.M., Gago, F., Egly, J.M., 2011. XPF-dependent DNA breaks and RNA polymerase II arrest induced by antitumor DNA interstrand crosslinking-mimetic alkaloids. Chem Biol 18, 988–99. https://doi.org/S1074-5521(11)00227-4 [pii] 10.1016/j.chembiol.2011.06.007

Kristensen, U., Epanchintsev, A., Rauschendorf, M.A., Laugel, V., Stevnsner, T., Bohr, V.A., Coin, F., Egly, J.M., 2013. Regulatory interplay of Cockayne syndrome B ATPase and stress-response gene ATF3 following genotoxic stress. Proceedings of the National Academy of Sciences of the United States of America 110, E2261-70. https://doi.org/10.1073/pnas.1220071110

Le May, N., Mota-Fernandes, D., Velez-Cruz, R., Iltis, I., Biard, D., Egly, J.M., 2010. NER factors are recruited to active promoters and facilitate chromatin modification for transcription in the absence of exogenous genotoxic attack. Mol Cell 38, 54–66. https://doi.org/S1097-2765(10)00211-X [pii] 10.1016/j.molcel.2010.03.004

Nagy, Z., Kalousi, A., Furst, A., Koch, M., Fischer, B., Soutoglou, E., 2016. Tankyrases Promote Homologous Recombination and Check Point Activation in Response to DSBs. PLoS Genet 12, e1005791. https://doi.org/10.1371/journal.pgen.1005791

Nieminuszczy, J., Broderick, R., Bellani, M.A., Smethurst, E., Schwab, R.A., Cherdyntseva, V., Evmorfopoulou, T., Lin, Y.-L., Minczuk, M., Pasero, P., Gagos, S., Seidman, M.M., Niedzwiedz, W., 2019. EXD2 Protects Stressed Replication Forks and Is Required for Cell Viability in the Absence of BRCA1/2. Molecular Cell 75, 605-619.e6. https://doi.org/10.1016/j.molcel.2019.05.026

Shanbhag, N.M., Rafalska-Metcalf, I.U., Balane-Bolivar, C., Janicki, S.M., Greenberg, R.A., 2010. ATM-dependent chromatin changes silence transcription in cis to DNA double-strand breaks. Cell 141, 970–81. https://doi.org/10.1016/j.cell.2010.04.038
