# Supplementary material for: Active mRNA degradation by EXD2 nuclease elicits recovery of transcription after genotoxic stress
Source: Nat Commun. 2023 Jan 20;14:341. doi: 10.1038/s41467-023-35922-5 (PMC9859823; doi:10.1038/s41467-023-35922-5)
Supplement: Supplementary file 2 — Reporting Summary [file 41467_2023_35922_MOESM2_ESM.pdf]

nature portfolio

Corresponding author(s): coin

Last updated by author(s): Dec 5, 2022

Reporting Summary

Nature Portfolio wishes to improve the reproducibility of the work that we publish. This form provides structure for consistency and transparency in reporting. For further information on Nature Portfolio policies, see our Editorial Policies and the Editorial Policy Checklist.

Statistics

For all statistical analyses, confirm that the following items are present in the figure legend, table legend, main text, or Methods section.

n/a	Confirmed	
☐	☒	The exact sample size (*n*) for each experimental group/condition, given as a discrete number and unit of measurement
☐	☒	A statement on whether measurements were taken from distinct samples or whether the same sample was measured repeatedly
☐	☒	The statistical test(s) used AND whether they are one- or two-sided<br>*Only common tests should be described solely by name; describe more complex techniques in the Methods section.*
☐	☒	A description of all covariates tested
☐	☒	A description of any assumptions or corrections, such as tests of normality and adjustment for multiple comparisons
☐	☒	A full description of the statistical parameters including central tendency (e.g. means) or other basic estimates (e.g. regression coefficient) AND variation (e.g. standard deviation) or associated estimates of uncertainty (e.g. confidence intervals)
☐	☒	For null hypothesis testing, the test statistic (e.g. *F*, *t*, *r*) with confidence intervals, effect sizes, degrees of freedom and *P* value noted<br>*Give P values as exact values whenever suitable.*
☒	☐	For Bayesian analysis, information on the choice of priors and Markov chain Monte Carlo settings
☒	☐	For hierarchical and complex designs, identification of the appropriate level for tests and full reporting of outcomes
☒	☐	Estimates of effect sizes (e.g. Cohen's *d*, Pearson's *r*), indicating how they were calculated

Our web collection on statistics for biologists contains articles on many of the points above.

Software and code

Policy information about availability of computer code

Data collection	no software was used for data collection
Data analysis	*Provide a description of all commercial, open source and custom code used to analyse the data in this study, specifying the version used OR state that no software was used.*

For manuscripts utilizing custom algorithms or software that are central to the research but not yet described in published literature, software must be made available to editors and reviewers. We strongly encourage code deposition in a community repository (e.g. GitHub). See the Nature Portfolio guidelines for submitting code & software for further information.

Data

Policy information about availability of data

All manuscripts must include a data availability statement. This statement should provide the following information, where applicable:

- Accession codes, unique identifiers, or web links for publicly available datasets
- A description of any restrictions on data availability
- For clinical datasets or third party data, please ensure that the statement adheres to our policy

All data from this paper are freely available

Human research participants

Policy information about studies involving human research participants and Sex and Gender in Research.

Reporting on sex and gender	N/A
Population characteristics	N/A
Recruitment	N/A
Ethics oversight	N/A

Note that full information on the approval of the study protocol must also be provided in the manuscript.

Field-specific reporting

Please select the one below that is the best fit for your research. If you are not sure, read the appropriate sections before making your selection.

☒ Life sciences ☐ Behavioural & social sciences ☐ Ecological, evolutionary & environmental sciences

For a reference copy of the document with all sections, see nature.com/documents/nr-reporting-summary-flat.pdf

Life sciences study design

All studies must disclose on these points even when the disclosure is negative.

Sample size	sample size is indicated for each experiment and was chosen to calculate statistical significant
Data exclusions	no data exclusion
Replication	replication is indicated, biological triplicates or technical tripmicates, all attempts were successful
Randomization	N/A no animal was used in this paper and therefore no randomization was necessary
Blinding	N/A no animal was used in this paper and therefore no blinding was necessary

Reporting for specific materials, systems and methods

We require information from authors about some types of materials, experimental systems and methods used in many studies. Here, indicate whether each material, system or method listed is relevant to your study. If you are not sure if a list item applies to your research, read the appropriate section before selecting a response.

Materials & experimental systems

n/a	Involved in the study	
☐	☒	Antibodies
☐	☒	Eukaryotic cell lines
☐	☐	Palaeontology and archaeology
☒	☐	Animals and other organisms
☒	☐	Clinical data
☒	☐	Dual use research of concern

Methods

n/a	Involved in the study	
☒	☐	ChIP-seq
☒	☐	Flow cytometry
☒	☐	MRI-based neuroimaging

Antibodies

Antibodies used	all antibodies used are described in Supplemental Table 1
Validation	Anti-Actin mouse monoclonal, Abcam ab 8245<br>https://www.abcam.com/gapdh-antibody-6c5-loading-control-ab8245.html<br>Validated by the company and following publication : Wang, H., et al., Cell Death Diff 13 :435 (2022)<br><br>Anti-CPD mouse monoclonal, Cosmo Bio CAC-NM-DND-001<br>https://www.cosmobiousa.com/products/anti-cpds-mab-clone-tdm-2

Validated by the company and following publication : Mori, T., et al., Photochem. Photobiol. 54, 225-232 (1991)

Anti-ATP5A mouse monoclonal, Proteintech 66037-1
https://www.ptglab.com/products/ATP5A1-Antibody-66037-1-Ig.htm
Validated by the company and following publication : Lu, YT., et al., Cell Death Dis, 9(6) :672 (2018)

Anti-EXD2 rabbit polyclonal, SIGMA, HPA00548
https://www.sigmaaldrich.com/FR/fr/product/sigma/hpa005848
Validated by the company and following publication : Biehs, R., et al., Molecular Cell, 65(4) :671-684 (2017)

Anti-Flag M2 mouse monoclonal, SIGMA, F1804
https://www.sigmaaldrich.com/FR/fr/product/sigma/f1804?gclsrc=aw.ds&gclid=EAIaIQobChMIv_mRn8-F_AIVkc3VCh0Y0wN1EAAYASAAEgJiyvD_BwE
Validated by the company and following publication : Srivastava, M., et al., Nature Communications, 6, 6253 (2015)

Anti-GAPDH mouse monoclonal, Abcam ab 8245
https://www.abcam.com/gapdh-antibody-6c5-loading-control-ab8245.html
Validated by the company and following publication : Wang, H., et al., Cell Death Diff 13 :435 (2022)

Anti-GFP rabbit polyclonal, Amsbio TP401
https://www.amsbio.com/rabbit-anti-gfp-pab-tp401
Validated by the company and following publication : Kosar, M., et al., Nature Communications, 12, 3937 (2021)

Anti-GFP mouse monoclonal, Roche 11814460001
https://www.sigmaaldrich.com/FR/fr/product/roche/11814460001?gclsrc=aw.ds&gclid=EAIaIQobChMItNGEgNuF_AIVh6ztCh1uaQljEAAYASAAEgL-hvD_BwE
Validated by the company and following publication : Chalfie, M., Science, 263, 802-805 (1994)

Anti-GST rabbit monoclonal, Abcam ab19256
https://www.abcam.com/gst-antibody-ab19256.htmlValidated by the company and following publication : Joset, A., J Cell Biol 188 :271-85 (2010).

Anti-gH2AX mouse monoclonal, Abcam ab22551
https://www.abcam.com/gamma-h2ax-phospho-s139-antibody-3f2-ab22551.html
Validated by the company and following publication : Ochs, F., Nature 574 :571-574 (2019)

Anti-Histone H3 rabbit polyclonal, Abcam ab1791
https://www.abcam.com/histone-h3-antibody-nuclear-marker-and-chip-grade-ab1791.html
Validated by the company and following publication : Yuan, L., NAR, 49 :98-113 (2021)

Anti-RPB1 mouse monoclonal, 7C2 IGBMC Home made
Validated following publication : Boulon, S., Mol Cell, 39(6) : 912-924

Anti-RPB1 rabbit polyclonal, Bethyl A300-653A
https://www.fortislife.com/products/primary-antibodies/rabbit-anti-rna-polymerase-ii-antibody/BETHYL-A300-653#dummy-cit
Validated following publication : Sharifnia, W., Nat Med 292-300 (2019)

Anti-TFIIEa mouse monoclonal, 2A1 IGBMC Home made
Validated following publication : Compe, E., Nature Communications, 10 :2084 (2019)

Anti-Vinculin mouse monoclonal, Sigma V9131
https://www.sigmaaldrich.com/FR/fr/sds/sigma/v9131
Validated following publication : Berico, P., Embo Rep, 22(9) 51683

Anti-XPA rabbit polyclonal, SCBT sc-853
https://www.scbt.com/fr/p/xpa-antibody-fl-273?requestFrom=search
Validated following publication : Fischer, J.M., The FEBS journal, 281 : 3625-41

Anti-(6-4PPs) mouse monoclonal, Clone 64M-2
https://www.cosmobiousa.com/products/anti-6-4pps-mab-clone-64m-2
Validated following publication : Yamamoto, A., et al., DNA Repair, 6, 649-657 (2007).

Eukaryotic cell lines

Policy information about cell lines and Sex and Gender in Research

Cell line source(s)	U-2 OS, Hela, CS1ANSV, CS1ANSV+CSB
Authentication	no authentification
Mycoplasma contamination	no contamination certified by our cell culture departement
Commonly misidentified lines (See ICLAC register)	no commonly misidentified lines used in this work

Palaeontology and Archaeology

Specimen provenance	*Provide provenance information for specimens and describe permits that were obtained for the work (including the name of the issuing authority, the date of issue, and any identifying information). Permits should encompass collection and, where applicable, export.*
Specimen deposition	*Indicate where the specimens have been deposited to permit free access by other researchers.*
Dating methods	*If new dates are provided, describe how they were obtained (e.g. collection, storage, sample pretreatment and measurement), where they were obtained (i.e. lab name), the calibration program and the protocol for quality assurance OR state that no new dates are provided.*

☐ Tick this box to confirm that the raw and calibrated dates are available in the paper or in Supplementary Information.

Ethics oversight	*Identify the organization(s) that approved or provided guidance on the study protocol, OR state that no ethical approval or guidance was required and explain why not.*

Note that full information on the approval of the study protocol must also be provided in the manuscript.

March 2021
